# SPARK‐ICA: Guided Frequency‐Resolved Dynamic Spatial ICA Reveals Fine‐Scale fMRI Network Dynamics

**DOI:** 10.1002/hbm.70662

**Published:** 2026-11-02

**Authors:** N. Behzadfar, A. Iraji, V. Calhoun

**Affiliations:** ^1^ Department of Electrical Engineering Na.C., Islamic Azad University Najafabad Iran; ^2^ Digital Processing and Machine Vision Research Center Na.C., Islamic Azad University Najafabad Iran; ^3^ Tri‐Institutional Center for Translational Research in Neuroimaging and Data Science (TReNDS) Georgia State University, Georgia Institute of Technology Atlanta Georgia USA

## Abstract

Fine‐scale spatiotemporal dynamics of functional brain networks are increasingly recognized as critical markers of transient connectivity and neuropsychiatric pathology. However, many existing dynamic fMRI methods primarily focus on temporal variability without explicitly resolving the spatial‐frequency content of network dynamics, potentially limiting characterization of subtle, high‐frequency spatial reconfigurations. To address this gap, we propose SPARK–ICA (Spatial–frequency Partitioned, Adaptive Reconstruction using Knowledge‐guided ICA), a frequency‐resolved dynamic spatial independent component analysis (ICA) framework that integrates NeuroMark template guidance with spatial frequency decomposition and group‐level ICA of time‐resolved, band‐specific spatial maps. Starting from template‐guided NeuroMark networks, we first estimate time‐varying spatial maps using a dynamic guided ICA approach, capturing subject‐specific network expression over time. Each dynamic spatial map is then decomposed into multiple spatial frequency bands using Fourier‐domain spatial filtering, isolating fine‐ to coarse‐scale spatial features at each time point. Band‐specific spatial maps are subsequently concatenated and subjected to a blind group‐level multi‐frequency ICA, yielding a compact latent representation in which each dynamic component is characterized by distinct spatial‐frequency signatures. These group‐level band‐resolved components are then used as references to estimate subject‐ and band‐specific time courses and maps via spatially constrained ICA, enabling frequency‐specific analysis of both long‐term correlations and short‐term temporal variability while preserving reproducibility across subjects. Using resting‐state fMRI data from schizophrenia (SZ) patients and healthy controls (HC), we evaluated both long‐term correlations and short‐term variability of band‐resolved dynamic time courses. For the group‐level analyses, we focused on a predefined subset of NeuroMark networks with well‐established involvement in SZ‐related dysconnectivity. Our results reveal frequency‐specific abnormalities in SZ, with enhanced long‐term connectivity within sensory and subcortical networks and pronounced short‐term alterations involving predominantly higher‐frequency spatial components, particularly in cerebellar and subcortical systems. These findings indicate that SZ‐related dysregulation is differentially expressed across spatial‐frequency and temporal scales, rather than reflecting uniform dysconnectivity. Together, this framework provides a unified approach for analyzing dynamic fMRI networks across spatial, temporal, and frequency dimensions, extending our previous NeuroMark‐DyFICA framework from a predominantly high‐frequency spatial representation to an explicitly frequency‐resolved characterization of dynamic network organization. By explicitly modeling spatial frequency–dependent dynamics, the proposed method provides a more resolved characterization of network alterations and offers a framework for identifying candidate markers of neuropsychiatric disorders.

## Graphical abstract

SPARK–ICA is a frequency‐resolved dynamic spatial ICA framework that decomposes time‐varying fMRI network maps into multiple spatial frequency bands. Applied to schizophrenia resting‐state data, it reveals complementary abnormalities in stable connectivity and rapid high‐frequency network interactions that are not captured by conventional dynamic connectivity analyses.

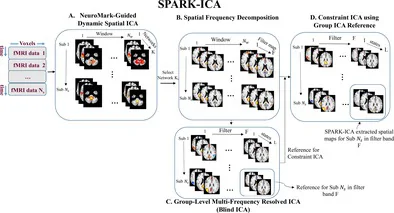

## 1 Introduction

Resting‐state functional magnetic resonance imaging (rs‐fMRI) has become a central tool for studying large‐scale brain organization, revealing consistent functional networks that reflect coordinated neural activity across distributed brain regions. Early work in this area largely relied on static functional connectivity, assuming temporal stationarity of network interactions across the scan duration. However, accumulating evidence suggests that functional brain networks exhibit not only temporal variability but also dynamic changes in their spatial organization, with transient reconfigurations in the spatial topography of networks even at rest (Hutchison et al. 2013; Calhoun et al. 2014; Iraji, Deramus, et al. 2019; Iraji, Fu, et al. 2019). These time‐resolved spatial maps, often referred to as the spatial chronnectome, capture dynamic patterns of segregation and integration across functional domains and provide complementary information to conventional temporal connectivity measures. Importantly, spatial dynamics have been shown to carry meaningful information about cognition and neuropsychiatric disorders (Iraji et al. 2020; Pusuluri et al. 2024). In schizophrenia (SZ), for example, spatially resolved dynamic analyses reveal atypical network overlap, disrupted transient interactions, and altered coupling, complementing temporal connectivity findings (Iraji et al. 2020; Pusuluri et al. 2024; Damaraju et al. 2014). Recent work has highlighted the hierarchical and multi‐dimensional nature of spatial dynamics. For example, within‐network and between‐network spatial fluctuations have been demonstrated using rs‐fMRI (Iraji, Deramus, et al. 2019; Iraji, Fu, et al. 2019), and conceptual reviews emphasize the importance of including spatial variability alongside temporal connectivity (Iraji et al. 2020). More recent studies show that such spatial dynamics are altered in neuropsychiatric disorders, including sex‐specific disruptions in schizophrenia (Iraji et al. 2024).

Dynamic functional connectivity (dFC) and its network analog, dynamic functional network connectivity (dFNC), are most commonly estimated using sliding‐window correlation approaches, in which connectivity is computed within overlapping temporal windows. While this strategy has provided valuable insights into temporal fluctuations, it does not fully capture the spatial reconfigurations of networks that occur over time. The choice of window length imposes an implicit temporal scale that may be suboptimal for fast or transient network changes (Leonardi and Van De Ville 2015; Lindquist et al. 2014). To address this limitation, some studies have systematically examined multiple window lengths or proposed time‐dependent/adaptive windowing strategies to better capture dynamics across multiple temporal scales (Leonardi and Van De Ville 2015; Preti et al. 2017). In addition, dynamic estimates may reflect a combination of genuine neural variability, noise, and smoothing effects, particularly when probing faster dynamics or shorter time scales (Preti et al. 2017; Shakil et al. 2016).

To complement temporal analyses, recent work has emphasized the frequency‐resolved connectivity. In particular, Faghiri et al. introduced the filter‐bank connectivity (FBC) framework, which estimates functional network connectivity across multiple temporal frequency bands rather than relying on a single window choice (filter) (Faghiri et al. 2021). This approach demonstrated that connectivity exhibits distinct frequency‐dependent patterns and revealed dynamic states that are not captured by conventional window‐based analyses. Importantly, FBC showed that SZ patients tend to occupy higher‐frequency connectivity states more frequently than healthy controls (HCs), highlighting the clinical relevance of frequency‐resolved connectivity analysis. Despite these advances, existing frequency‐based methods almost exclusively focus on the temporal frequency of connectivity estimates, while largely neglecting the spatial frequency characteristics of the underlying functional networks themselves.

Spatial organization is a fundamental aspect of brain networks, spanning multiple network sizes, from coarse, smoothly varying patterns to fine‐grained, localized features (Iraji et al. 2022; Iraji et al. 2023). Conventional independent component analysis (ICA) of fMRI data tends to capture networks with low spatial frequencies, favoring smooth, spatially extended network representations (Calhoun and Adali 2012; Smith et al. 2012). However, growing evidence suggests that high‐spatial‐detail variations within networks may reflect transient reconfigurations, localized engagement, or pathological alterations that are not adequately captured by low‐frequency‐dominated representations (Behzadfar et al. 2025). High spatial‐frequency components are often attenuated or discarded during preprocessing, despite their potential relevance for characterizing subtle and short‐lived network dynamics.

Template‐guided ICA frameworks such as NeuroMark provide a powerful solution for studying brain networks in a reproducible and comparable manner across subjects and studies (Du 2020). By constraining ICA with replicable network templates, NeuroMark enables subject‐specific estimation of functional networks within a common latent space, facilitating group‐level inference while preserving individual variability. Building on this framework, we previously introduced NeuroMark‐DyFICA, a dynamic frequency‐informed ICA approach that integrates sliding‐window analysis, high‐pass spatial filtering, and group‐level ICA to characterize fine‐scale spatiotemporal variations in functional networks (Behzadfar et al. 2026). NeuroMark‐DyFICA demonstrated sensitivity to subtle spatial shifts and transient network reconfigurations in both controlled simulations and resting‐state fMRI data from SZ patients and healthy controls. In the same fBIRN dataset analyzed in the present study, NeuroMark‐DyFICA identified abnormalities in the distribution and coordination of fine‐scale dynamic states, including altered state prevalence in auditory, visual/fusiform, middle frontal, and cerebellar networks and altered convergence among dynamic states in visual/fusiform, middle frontal, and default‐mode networks (Behzadfar et al. 2026). However, NeuroMark‐DyFICA represented the retained high‐spatial‐frequency content as a single high‐frequency representation and characterized its dynamics primarily through state‐density and convergence/divergence measures. Consequently, it did not resolve whether different spatial‐frequency scales contribute differently to stable versus rapidly fluctuating network interactions.

In the present work, we extend these ideas by explicitly decomposing dynamic network maps into multiple spatial‐frequency bands and subsequently estimating frequency‐resolved dynamic components. SPARK–ICA (Spatial–frequency Partitioned, Adaptive Reconstruction using Knowledge‐guided ICA) therefore retains the fine‐scale spatial sensitivity of NeuroMark‐DyFICA while adding spatial‐frequency resolution to the dynamic representation. Rather than asking only whether fine‐scale spatial dynamics are altered, the present framework asks how these alterations are distributed across spatial scales and whether their spatial‐frequency organization differs between stable and rapidly fluctuating network interactions. The resulting frequency‐resolved components are reconstructed at the subject level, enabling direct comparison of long‐term and short‐term dynamics across spatial‐frequency bands within a common NeuroMark‐guided framework.

To evaluate the proposed framework, we examine both long‐term and short‐term correlation structures of the reconstructed time courses, capturing stable connectivity patterns as well as rapid fluctuations in network interactions. By comparing these measures between SZ patients and HCs, we aim to determine whether disease‐related alterations are differentially expressed across spatial‐frequency and temporal scales. While the proposed SPARK–ICA framework is general and applicable to the full NeuroMark atlas, in the present study we focus on a subset of networks that have been most consistently implicated in SZ‐related dysconnectivity. This targeted analysis allows us to illustrate the utility of spatial‐frequency–resolved dynamics in well‐characterized systems, while maintaining a clear link to prior neurobiological findings. Together, this work advances dynamic fMRI analysis by unifying spatial frequency decomposition, reproducible ICA modeling, and time‐resolved spatial dynamics, offering a novel perspective on fine‐scale spatiotemporal organization of functional brain networks.

## 2 Methods

The proposed SPARK–ICA approach is a guided frequency‐resolved dynamic spatial ICA framework designed to characterize fine‐scale spatiotemporal network dynamics in resting‐state fMRI. As illustrated in Figure 1, the method comprises four key stages, integrating template‐guided dynamic network estimation, spatial frequency decomposition, group‐level ICA modeling, and spatially constrained ICA into a unified pipeline.

**FIGURE 1:**
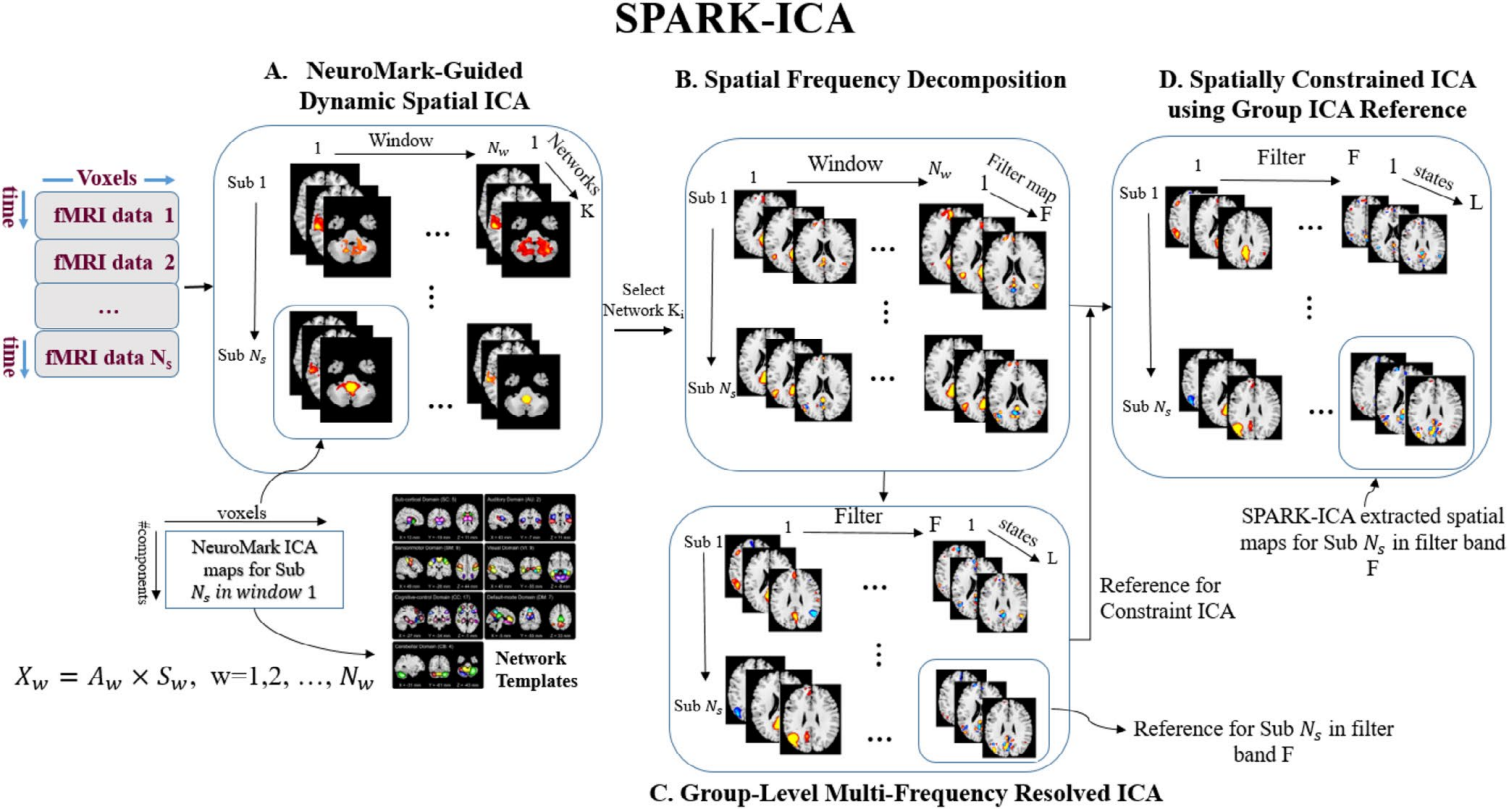
Overview of the SPARK–ICA framework for frequency‐resolved dynamic spatial network analysis. Starting from resting‐state fMRI data, NeuroMark‐guided dynamic spatial ICA is applied within sliding windows to estimate time‐varying spatial network maps. Each dynamic map is then decomposed into multiple spatial frequency bands using Fourier‐domain spatial filtering. Band‐specific maps are concatenated across time and subjects and analyzed using group‐level frequency‐resolved ICA, yielding spatial components characterized by distinct spatial‐frequency signatures. These group‐level components are subsequently used as references to reconstruct subject‐ and band‐specific dynamic spatial maps and time courses, enabling analysis of network dynamics across coarse‐to‐fine spatial frequency bands.

Starting from NeuroMark‐defined network templates, time‐varying spatial maps are estimated for each subject using a dynamic guided ICA approach (Iraji et al. 2024). This step captures the subject‐specific temporal evolution of canonical brain networks while constraining the decomposition with prior template information. Each dynamic spatial map is then decomposed into multiple spatial frequency bands using Fourier‐domain spatial filtering (in this work, four spatial frequency bands). This decomposition separates coarse‐to‐fine spatial features at each time point, allowing the analysis of network dynamics across different spatial scales. Band‐resolved spatial maps are subsequently concatenated across time and frequency and analyzed using group‐level ICA. Although all frequency bands are included in the concatenation, the resulting components often load predominantly on specific frequency bands, providing a latent representation of frequency‐biased patterns shared across subjects. These group‐level components are then used as references to reconstruct subject‐ and band‐specific time courses via spatially constrained ICA, enabling the quantitative assessment of temporal fluctuations within subjects and ensuring that the observed dynamic patterns reflect within‐subject network dynamics rather than only between‐subject differences. This approach allows the evaluation of network interactions across spatial, temporal, and frequency dimensions.

### 2.1 NeuroMark‐Guided Dynamic Spatial ICA

In the first stage of the SPARK–ICA framework, we estimate time‐varying spatial brain networks using a dynamic extension of the NeuroMark ICA model. The standard NeuroMark approach provides reproducible intrinsic connectivity network templates derived from large‐scale resting‐state fMRI datasets through group ICA, followed by extensive replication and stability analyses (Du 2020). These templates define a common functional network space that enables consistent component identification across subjects and studies.

To capture temporal variability in network organization, the NeuroMark‐guided ICA model is applied within overlapping sliding windows of the fMRI time series. For each window, ICA decomposition incorporates the NeuroMark templates as spatial reference priors using a multi‐objective optimization ICA with reference (MOO‐ICAR) algorithm. This provides soft spatial constraints that promote correspondence with the template networks while still allowing window‐ and subject‐specific deviations in spatial topography (Iraji et al. 2020; Iraji et al. 2024). This preserves correspondence across windows and subjects without enforcing identical spatial maps.

Formally, the decomposition for each window w is expressed as:

(1) $$X_{w}=A_{w}\times S_{w},w=1,\;2,\cdots ,N_{w}$$

where $X_{w}$ denotes the fMRI data matrix within window w, $S_{w}$ represents the window‐specific spatial maps constrained by the NeuroMark templates, and $A_{w}$ contains the corresponding mixing time courses. This procedure yields a sequence of dynamic spatial maps for each subject, reflecting how template‐guided functional networks evolve over time (Figure 1A).

In SPARK–ICA, the resulting dynamic spatial maps serve as the input to the subsequent spatial‐frequency decomposition stage.

### 2.2 Spatial Frequency Decomposition of Dynamic Maps

Following NeuroMark‐guided dynamic network estimation, the resulting dynamic spatial maps are decomposed into multiple spatial frequency bands to characterize both coarse‐scale and fine‐scale variations in brain network organization over time. This decomposition is motivated by the observation that canonical functional networks are largely dominated by smooth, low spatial‐frequency structure, whereas transient, localized, or pathological alterations in these networks may preferentially manifest at higher spatial frequencies and remain obscured in broadband spatial representations.

Spatial frequency analysis is performed independently for each NeuroMark‐defined network. For a given NeuroMark network, its dynamic spatial maps are analyzed separately across all sliding windows and subjects. This network‐wise strategy preserves interpretability at the level of predefined intrinsic connectivity networks and avoids mixing spatial‐frequency content across distinct functional systems.

For each subject sb, window w, and selected network k, let $S_{\mathrm{sb},w}^{\left(k\right)}\left(r\right)$, where r=(x,y,z), denote the corresponding three–dimensional spatial map, which serves as the input to the spatial–frequency analysis. Each spatial map is transformed into the three–dimensional Fourier domain as

(2) $$F_{\mathrm{sb},w}^{\left(k\right)}\left(\omega \right)=F\left\{S_{\mathrm{sb},w}^{\left(k\right)}\left(r\right)\right\}$$

where $\omega =\left(\omega_{x},\omega_{y},\omega_{z}\right)$ denotes the three‐dimensional spatial–frequency coordinates. The normalized radial spatial frequency is then defined as:

(3) $$\rho \left(\omega \right)=\frac{\sqrt{\omega_{x}^{2}+\omega_{y}^{2}+\omega_{z}^{2}}}{\underset{\omega }{\max}\left(\sqrt{\omega_{x}^{2}+\omega_{y}^{2}+\omega_{z}^{2}}\right)}$$

which provides a normalized measure ranging from the DC component (ρ=0) to the Nyquist spatial frequency (ρ=1).

Based on the normalized radial frequency representation $\rho \left(\omega \right)$, each spatial map is decomposed using a multi–band spatial filter bank consisting of B non–overlapping frequency bands. These bands span progressively finer spatial scales, ranging from smooth, large–scale network structure to localized, high–frequency spatial variations.

For each band b, a smooth Butterworth–style band–pass filter is constructed to avoid sharp spectral cutoffs and the associated ringing artifacts. Given lower and upper cutoff frequencies $\rho_{b}^{\mathrm{low}}$ and $\rho_{b}^{\text{high}}$, the band–pass transfer function is defined as the product of low–pass and high–pass components:

(4) $$\;H_{b}\left(\rho \right)=\frac{1}{1+{\left(\frac{\rho }{\rho_{b}^{\text{high}}}\right)}^{2n}}\cdot \frac{1}{1+{\left(\frac{\rho_{b}^{\mathrm{low}}}{\rho }\right)}^{2n}}\;$$

where n denotes the filter order and controls the smoothness of the transition between passband and stopband. The band–limited spatial map is then obtained by applying the filter in the Fourier domain and transforming back to the spatial domain via the inverse Fourier transform:

(5) $$\begin{array}{cccc} & S_{\mathrm{sb},w}^{\left(k,b\right)}\left(r\right)=F^{-1}\left\{F_{s,w}^{\left(k\right)}\left(\omega \right)\;H_{b}\left(\rho \left(\omega \right)\right)\right\} & & \end{array}$$

To ensure spatial consistency across sliding windows and subjects, each band‐limited spatial map is voxel‐wise multiplied by a window‐specific binary brain mask derived within the NeuroMark pipeline. The mask restricts the analysis to voxels consistently represented across subjects within each window. Following masking, each band‐limited map is voxel‐wise weighted by the absolute value of the corresponding group‐level NeuroMark spatial template to preserve correspondence with the canonical network structure while allowing frequency‐specific modulation. This weighting is performed in the spatial domain and does not alter the spatial‐frequency decomposition; rather, it emphasizes frequency‐specific features aligned with the target network (Mirzaeian et al. 2025).

For each subject, window, network, and spatial frequency band, this procedure produces frequency‐resolved dynamic spatial maps. These maps constitute the input to the subsequent group‐level frequency‐resolved ICA stage (Figure 1B).

### 2.3 Group‐Level Frequency‐Resolved ICA

Following spatial frequency decomposition of the dynamic NeuroMark‐guided spatial maps, a blind group‐level frequency‐resolved ICA is performed to identify latent spatial patterns shared across subjects, windows, and spatial frequency bands. The resulting components provide a common latent representation of frequency‐resolved dynamic spatial variability within each NeuroMark network.

For a given NeuroMark network k, each subject sb, has W sliding windows and F spatial frequency bands, yielding a set of band‐limited spatial maps $S_{w}^{\left(k,\mathrm{sb},f\right)}\left(r\right)$.

For each subject, the W×F spatial maps are vectorized and concatenated to form a subject‐specific matrix:

(6) $$X^{\left(k,\mathrm{sb}\right)}=\left[\begin{array}{c} \mathrm{vec}\left(S_{1}^{\left(k,\mathrm{sb},1\right)}\right)\\ \vdots \\ \mathrm{vec}\left(S_{W}^{\left(k,\mathrm{sb},F\right)}\right) \end{array}\right]\in R^{\left(W,F\right)\times V}$$

where each row corresponds to one window‐frequency observation and V is the number of voxels. Subject‐level matrices are then vertically concatenated across subjects to construct a population‐level dataset used for ICA.

Prior to dimensionality reduction, the concatenated data matrix is mean‐centered and variance‐normalized across observations (across window‐frequency instances) to reduce scale differences among observations and spatial frequency bands and to improve the stability of subsequent PCA and ICA estimation.

Dimensionality reduction is then performed using a single group‐level PCA, retaining a fixed number of components that capture the majority of variance in the concatenated data. Group‐level PCA is used to preserve a common latent space across subjects, enabling extraction of shared spatial‐frequency patterns. ICA is subsequently applied to the reduced data to decompose the concatenated spatial maps into maximally independent components:

(7) $$Z^{\left(k\right)}\approx A^{\left(k\right)}S^{\left(k\right)}$$

where $Z^{\left(k\right)}$ denotes the PCA‐reduced representation of the concatenated spatial maps for NeuroMark network k. The matrix $S^{\left(k\right)}\in R^{L\times V}$ contains L group‐level spatial components (rows), each representing a latent spatial pattern associated with the selected NeuroMark network and characterized by a distinct spatial‐frequency signature. The number of components L is determined using the elbow criterion applied to the PCA scree curve, providing a balance between variance representation and dimensional compactness while avoiding overfitting. The mixing matrix $A^{\left(k\right)}$ captures how these group‐level spatial components combine to explain the PCA‐reduced observations, which correspond to concatenated window–frequency samples across subjects. At this stage, the ICA decomposition identifies shared spatial‐frequency patterns across the population but does not directly provide subject‐ or window‐specific component expression.

Because the input to group‐level ICA consists of spatial maps organized by spatial frequency band, the resulting components encode frequency‐resolved spatial structure at the group level. In this way, the ICA decomposition separates not only distinct spatial configurations but also their frequency‐dependent manifestations over time.

The resulting group‐level components serve as spatial references for the subsequent subject‐ and band‐specific reconstruction. They provide a common spatial basis that preserves component correspondence while allowing individual and frequency‐specific variability to be quantified.

### 2.4 Spatially Constrained ICA for Subject‐ and Band‐Specific Time Course Reconstruction

Following group‐level frequency‐resolved ICA (Figure 1C), subject‐ and band‐specific network time courses are reconstructed using a spatially constrained ICA framework, in which the group‐level spatial components serve as fixed spatial references. This stage links the shared frequency‐resolved spatial representations identified at the group level to subject‐specific dynamic expressions while preserving component correspondence across subjects, spatial frequency bands, sliding windows, and NeuroMark networks, as illustrated in Figure 1D.

For a given NeuroMark network k, the group‐level analysis yields a set of L spatial components:

(8) $$S^{\left(k\right)}=\left\{s^{\left(k,1\right)},\cdots ,s^{\left(k,L\right)}\right\}\in R^{L\times V}$$

where V is the number of voxels in the network. These components serve as fixed spatial constraints in the subsequent reconstruction. For each subject sb and spatial frequency band f, the input to constrained ICA is the matrix of W dynamic spatial maps:

(9) $$X_{w}^{\left(\mathrm{sb}.f\right)}\in R^{W\times V}$$

where each row corresponds to a spatial map from a sliding window in that band. Under the spatially constrained model, the dynamic spatial maps are expressed as a linear combination of the group‐level components:

(10) $$X_{w}^{\left(\mathrm{sb},f\right)}\approx A_{w}^{\left(\mathrm{sb},f\right)}S^{\left(k\right)}$$

where $A^{\left(\mathrm{sb}.f\right)}\in R^{W\times L}$ contains the subject‐ and band‐specific time courses of the L components across W sliding windows. The time courses are estimated by minimizing the reconstruction error while keeping the group‐level spatial components fixed:

(11) $${\hat{A}}^{\left(\mathrm{sb},f\right)}=\arg\min_{A}{\left\|X^{\left(\mathrm{sb},f\right)}-AS^{\left(k\right)}\right\|}_{F}^{2}$$

This constraints optimization admits a closed‐form solution via least‐squares projection,

(12) $${\hat{A}}^{\left(\mathrm{sb},f\right)}=X^{\left(\mathrm{sb},f\right)}\;S^{\left(k\right)⊤}{\left(S^{\left(k\right)}S^{\left(k\right)⊤}\right)}^{-1}$$

This spatially constrained reconstruction is performed independently for each subject sb and frequency band f. For each NeuroMark network k, the total number of reconstruction is:

(13) $$N_{\text{runs}}=N_{s}\times F$$

where $N_{s}$ denotes the number of subjects and F the number of spatial frequency bands. This procedure yields frequency‐resolved dynamic component time courses for each subject and network, enabling direct comparison across subjects and bands while capturing individual variability in temporal expression.

## 3 Analysis of fMRI Data

We analyzed resting‐state fMRI data from 131 SZ patients and 141 HCs obtained from the Phase III Functional Biomedical Informatics Research Network (fBIRN) dataset (Potkin and Ford 2009). The analyzed cohort corresponds to a subset of the full fBIRN sample that met the methodological requirements of the present analysis, including consistent time‐series length across subjects. Detailed acquisition procedures have been reported previously (Damaraju et al. 2014); here we summarize the key steps relevant to the present study for clarity. Functional images were acquired across seven imaging sites using 3 T MRI scanners with a gradient‐echo echo‐planar imaging (EPI) sequence (field of view: 220 × 220 mm, matrix size: 64 × 64, TR = 2 s, TE = 30 ms, flip angle = 77°, 162 volumes, 32 axial slices with 4 mm thickness and 1 mm gap). Participants were instructed to remain still with eyes closed during scanning. Preprocessing was performed using SPM12 (http://www.fil.ion.ucl.ac.uk/spm/) and included slice‐timing correction, rigid‐body motion correction, and spatial normalization to the Montreal Neurological Institute (MNI) space using an EPI template. The fMRI data were resampled to 3 × 3 × 3 mm^3^ isotropic voxels during spatial normalization. To mitigate inter‐site variability in spatial smoothness, all data were spatially smoothed to 6 mm full width at half maximum (FWHM) using AFNI's Blur to FWHM algorithm, thereby reducing differences in effective spatial smoothness across sites. This preprocessing pipeline is consistent with prior fBIRN analyses (Damaraju et al. 2014). Following preprocessing, subject‐specific fMRI time series were segmented into overlapping temporal windows to capture time‐varying network structure. Each window comprised 60 consecutive time points (approximately 2 min, given TR = 2 s), with adjacent windows shifted by 2 time points, resulting in 49 windows per subject. The selected window length reflects a trade‐off between temporal resolution and estimation reliability: shorter windows can capture faster fluctuations but lead to noisier and less stable ICA estimates, whereas longer windows provide more robust spatial estimates at the cost of smoothing rapid transitions. A window length of approximately 1–2 min has been widely adopted in prior dynamic connectivity and dynamic ICA studies as a practical compromise (Behzadfar et al. 2026; Allen et al. 2014). The stride of 2 time points was chosen to balance increasing temporal sensitivity with computational feasibility. Although a stride of 1 would yield finer sampling, it would also nearly double the number of windows with substantial redundancy due to strong overlap between adjacent windows, resulting in limited Supporting Information but significantly increased computational burden. This configuration has been successfully used in our prior dynamic NeuroMark work and enables stable estimation of time‐resolved spatial networks with consistent correspondence across subjects.

Dynamic network estimation was performed using template‐guided NeuroMark ICA, implemented in the GIFT toolbox at http://trendscenter.org/software/gift (Calhoun et al. 2001; Iraji et al. 2021; Calhoun, n.d.). NeuroMark incorporates spatial priors derived from large, independent resting‐state datasets to ensure reproducible identification of intrinsic connectivity networks across subjects and studies. For each window and subject, spatially constrained ICA was applied using the MOO‐ICAR algorithm, guided by the NeuroMark_fmri_1.0 template (template available at https://trendscenter.org/data/) (Du 2020). This procedure yielded 53 intrinsic connectivity networks per window, preserving correspondence across time and subjects while allowing window‐specific spatial variability.

To explicitly characterize spatial‐scale–dependent dynamics, we selected NeuroMark‐defined networks and decomposed their dynamic spatial maps into multiple spatial frequency bands. For each subject, window, and selected network, the corresponding 3D spatial map was transformed into the Fourier domain and filtered using a multi‐band spatial filter bank. We restricted the analysis to the range 0.01–0.25 cycles per voxel, corresponding to approximately 0.0033–0.0833 cycles/mm for the 3 × 3 × 3 mm^3^ isotropic voxels used after spatial normalization. Equivalently, this frequency range corresponds to spatial wavelengths of approximately 300–12 mm. These conversions provide a physical‐scale interpretation of the spatial‐frequency bands in millimeter units. Importantly, these spatial‐frequency representations are derived from data that were spatially smoothed to 6 mm FWHM during preprocessing. Therefore, the nominal 3 mm voxel size should not be interpreted as the effective spatial resolution of the preprocessed data. Spatial smoothing attenuates fine spatial variations and may reduce the representation of the highest spatial frequencies, particularly in B3 and B4. Accordingly, these higher‐frequency bands should be interpreted as residual fine‐scale spatial variations in the preprocessed data rather than as fully resolved anatomical structures at the nominal voxel scale. Because the effective spatial resolution of the preprocessed fMRI data is determined not only by the voxel dimensions but also by the imaging point‐spread function and the applied 6 mm FWHM spatial smoothing, higher spatial frequencies are progressively attenuated and may be more susceptible to noise and partial‐volume effects (Kim et al. 2020; Logothetis 2016). Very low spatial frequencies (near DC) primarily reflect broad intensity bias and slow spatial drifts rather than localized network structure. Filtering out these near‐DC components suppresses the influence of large‐scale intensity gradients and global bias fields, which are typically treated as nuisance variation in spatial analysis and are commonly reduced by high‐pass filtering (Smith et al. 2013; Liu et al. 2017). To examine spatial organization at progressively finer spatial scales, this range was partitioned into four non‐overlapping radial spatial‐frequency bands:

- Band 1 (B1): 0.0100−0.0625 cycles/voxel (0.0033–0.0208 cycles/mm; approximately 48–300 mm wavelength)
- Band 2 (B2): 0.0625−0.1250 cycles/voxel (0.0208–0.0417 cycles/mm; approximately 24–48 mm wavelength)
- Band 3 (B3): 0.1250−0.1875 cycles/voxel (0.0417–0.0625 cycles/mm; approximately 16–24 mm wavelength)
- Band 4 (B4): 0.1875−0.2500 cycles/voxel (0.0625–0.0833 cycles/mm; approximately 12–16 mm wavelength).

These boundaries were selected to provide approximately equal logarithmic spacing across the usable spectrum, ensuring balanced resolution of coarse, intermediate, and fine scale spatial features, while maintaining numerical stability of band‐pass filtering. Spatial frequency filtering was performed in the Fourier domain using smooth Butterworth‐style band‐pass filters with order n=8. Butterworth filters are commonly used in spatial frequency analysis because they provide a maximally flat passband and smooth transitions between frequency bands, thereby reducing ringing artifacts relative to ideal (brick‐wall) filters while still allowing effective band separation. The trade‐off between sharper frequency separation and suppression of spatial artifacts as a function of filter order has been well documented in the image processing literature (Gonzalez 2009; Perfetto et al. 2020). In the present work, a moderate‐to‐high filter order (n=8) was selected empirically to achieve sufficient separation among the four spatial frequency bands while avoiding excessive ringing or numerical instability, consistent with established principles of spatial frequency filtering in image analysis… Each band‐limited map was transformed back to the spatial domain, masked to brain tissue, and voxel‐wise weighted by the absolute value of the corresponding NeuroMark group template to preserve network correspondence while allowing frequency‐specific modulation. This procedure generated frequency‐resolved dynamic spatial representations for each network, window, and subject.

Band‐resolved dynamic spatial maps obtained from all subjects and windows were concatenated and entered into a group‐level ICA to identify latent components representing shared spatial–frequency dynamics. Prior to ICA, dimensionality reduction was performed using PCA, retaining components that preserved maximal variance. For each NeuroMark template network, the model order of the subsequent ICA decomposition was determined from the corresponding PCA scree curve using a 95% cumulative explained‐variance criterion. This procedure resulted in a model order of six components for each of the NeuroMark networks analyzed in the present study. The model order was determined independently for each network based on its PCA variance structure rather than being fixed a priori. Supplementary Figure S1 shows the PCA scree curve and the selected six‐component model order for NeuroMark‐49 as an illustrative example; the same model‐order selection procedure was applied independently to the other analyzed NeuroMark networks. This data‐driven procedure provides a balance between variance preservation and model compactness while maintaining interpretability of the resulting components. Group ICA was then performed using the Infomax algorithm, yielding frequency‐resolved dynamic components with distinct spatial signatures. These group‐level components define a common latent representational space that enables direct comparison across subjects and supports subsequent reconstruction of subject‐ and band‐specific time courses.

To assess the reproducibility of the group‐level ICA decomposition, we evaluated component stability using the ICASSO framework as implemented in the GIFT toolbox (Himberg et al. 2004). ICASSO assesses the stability of independent components by comparing ICA solutions obtained from repeated runs with different random initializations. The group‐level ICA decomposition was repeated using different initializations, and the resulting components were clustered according to their spatial similarity. Component stability was quantified using the ICASSO stability index (Iq), with higher Iq values indicating more compact and reproducible component clusters across runs. This analysis was used to assess the robustness of the latent frequency‐resolved components to ICA initialization.

To obtain subject‐level dynamic representations, the group‐level frequency‐resolved components were used as spatial references in a spatially constrained ICA reconstruction step. For each subject and frequency band, we estimated ICA time courses that best explained the observed band‐limited spatial maps while maintaining correspondence with the group‐level components. This reconstruction yielded subject‐ and band‐specific dynamic time courses that preserve reproducibility across subjects while retaining individual variability.

Dynamic properties of the reconstructed time courses were quantified using complementary evaluation metrics capturing both stable and transient aspects of network organization. Specifically, we computed long‐term correlation structure, reflecting persistent coordination among frequency‐resolved components across the full scan duration, and short‐term correlation variability, which captures rapid fluctuations in component interactions across shorter temporal segments. Group differences were assessed using two‐sample statistical testing with false discovery rate (FDR) correction to control for multiple comparisons.

### 3.1 Statistical Analysis

Before computing temporal metrics on the reconstructed frequency‐resolved time courses, we focused our analysis on a subset of six NeuroMark networks that have been repeatedly implicated in SZ‐related dysconnectivity. These networks were selected a priori based on extensive prior evidence demonstrating abnormalities in thalamocortical communication and subcortical integration (Woodward et al. 2012; Anticevic et al. 2014; Giraldo‐Chica and Woodward 2017), alterations in visual and fusiform cortical processing (Iwabuchi and Palaniyappan 2017; Silverstein and Keane 2011; Maher et al. 2019; He et al. 2024), disruptions within the default‐mode network (DMN) (Whitfield‐Gabrieli and Ford 2012; Sasabayashi et al. 2023), aberrant auditory/temporal network activity linked to hallucinations (Williams et al. 2025; Alhazmi 2025; Zhang et al. 2025), impaired frontal executive control circuits (Jiang et al. 2019), and cerebellar dysregulation influencing cognitive integration (Forlim et al. 2024; Kim 2000; Park et al. 2021). Importantly, these same functional domains have also been shown to exhibit altered within‐ and between‐network spatial dynamics in schizophrenia, reflecting abnormal time‐varying patterns of segregation and integration even at rest, as demonstrated in prior spatial chronnectome studies (Iraji, Deramus, et al. 2019; Iraji, Fu, et al. 2019). Thus, focusing on these networks enables a hypothesis‐driven evaluation of whether schizophrenia manifests network‐specific alterations in temporal coordination that are differentially expressed across spatial frequency bands, while leveraging NeuroMark's reproducible network definitions as a stable reference framework. The underlying SPARK–ICA methodology itself remains fully applicable to the complete NeuroMark atlas.

To characterize and quantify how spatial–frequency–resolved dynamic networks evolve over time and differ between groups, we quantified complementary aspects of temporal organization using two dynamic evaluation metrics: (i) long‐range temporal correlation structure, reflecting sustained coordination patterns across the full scan duration, and (ii) short‐term temporal variability, reflecting rapid transient fluctuations in network interactions occurring over shorter time scales. Together, these measures capture both persistent and moment‐to‐moment aspects of neural dynamics, enabling a more complete assessment of frequency‐dependent network behavior in SZ.

All metrics were computed using the subject‐ and band‐specific dynamic time courses reconstructed in the previous stage. For each subject, time courses were concatenated across selected NeuroMark networks, ICA components, and spatial frequency bands, yielding a multivariate dynamic representation of network activity. Let $N_{\mathrm{net}}$ denote the number of selected NeuroMark networks, $N_{\text{comp}}$ the number of group‐level ICA components retained per network, and $N_{\text{band}}$ the number of spatial frequency bands. Let, $A^{\left(s\right)}\left(t\right)\in R^{D}$, denote the vector of reconstructed ICA time courses at time t for subject s, where, $D=N_{\mathrm{net}}\times N_{\text{comp}}\times N_{\text{band}}$, is the total number of band‐resolved dynamic components. Concatenating these time courses across sliding windows produces a matrix:

(14) $$A^{\left(s\right)}={\left[A^{\left(s\right)}\left(1\right)\;A^{\left(s\right)}\left(2\right)\cdots A^{\left(s\right)}\left(T\right)\right]}^{⊤}\in R^{T\times D}$$

which serves as the basis for all subsequent dynamic analyses.

Long‐range temporal correlations quantify stable statistical dependencies among dynamic components over the entire scan duration. For each subject, we computed the Pearson correlation matrix across all concatenated time points,

(15) $$C_{\text{long}}^{\left(s\right)}=\text{corr}\left(A^{\left(s\right)}\right)$$

where $C_{\text{long}}^{\left(s\right)}\in R^{D\times D}$ captures pairwise relationships between band‐resolved components.

This measure reflects persistent coupling patterns that are maintained across time, integrating information from both low‐ and high‐frequency spatial components. Group‐level mean correlation matrices were obtained by averaging subject‐specific matrices within each group, providing a summary of stable network organization.

While long‐term correlations capture overall stability of network interactions, they do not reflect how rapidly interactions fluctuate over time. To quantify temporal instability and transient reconfigurations, we assessed short‐term variability of correlation structure using a sliding‐segment approach.

For each subject, the concatenated dynamic time courses were partitioned into consecutive, non‐overlapping short segments of length M windows. In this study, M was set to 6 windows, yielding a total of Q=[W/M] segments per subject, where W is the total number of sliding windows. For each segment i=1,2.⋯,Q, a segment‐wise correlation matrix was computed as:

(16) $$C_{i}^{\left(s\right)}=\text{corr}\left(A^{\left(s\right)}\left(t_{i}:t_{i}+M-1\right)\right)$$

where $A^{\left(s\right)}\left(t\right)$ denotes the multivariate vector of reconstructed band‐resolved time courses for subject s.

The choice of M=6 windows provides a short‐term temporal scale that is sufficiently brief to capture rapid fluctuations in network coordination while retaining enough samples within each segment for stable correlation estimation, given the strong overlap between adjacent sliding windows.

Short‐term variability was then quantified as the element‐wise standard deviation of the segment‐wise correlation matrices across segments:

(17) $$V_{\text{short}}^{\left(s\right)}=\mathrm{std}\left(C_{1}^{\left(s\right)},\cdots ,C_{Q}^{\left(s\right)}\right)$$

Higher values indicate greater temporal instability, reflecting rapid switching or fluctuating coordination between components, whereas lower values suggest more consistent short‐term dynamics.

For both metrics, subject‐level matrices were compared between SZ patients and HCs using two‐sample *t*‐tests applied to each unique matrix entry. To control for multiple comparisons, statistical significance was assessed using a single global FDR correction applied to the lower triangular portion of each matrix, ensuring consistent control of false positives across all component pairs.

Effect directionality was retained via the sign of the corresponding t‐statistics, allowing interpretation of whether group differences reflected increased or decreased coupling or variability.

Together, these two metrics provide a complementary view of dynamic network organization. Long‐term correlations reflect stable, trait‐like coordination patterns, while short‐term variability captures moment‐to‐moment flexibility and transient reconfigurations. Importantly, because these measures are computed on spatial–frequency–resolved dynamic time courses, they are sensitive to alterations that may preferentially occur at specific spatial scales, extending beyond conventional broadband or window‐based dynamic connectivity analyses.

To examine the clinical relevance of dynamic functional connectivity, we evaluated associations between connectivity‐derived temporal metrics and clinical measures within the SZ group. Clinical variables included speed of processing, working memory (WM), and symptom severity assessed using the Positive and Negative Syndrome Scale, focusing on positive (PANSS‐P) and negative (PANSS‐N) symptom scores.

For each subject, connectivity metrics derived from both long‐term and short‐term dynamic analyses were correlated with clinical measures using Spearman's rank correlation, chosen to account for potential non‐normality in both imaging and behavioral variables. Associations were evaluated separately for long‐term and short‐term dynamics.

To control for multiple comparisons across components, spatial frequency bands, and networks, FDR correction was applied at q<0.05. The number of FDR‐significant associations was summarized for each clinical measure and temporal scale. Additional analyses examined the distribution of significant associations across spatial frequency bands, ICA components, and functional networks to characterize the multiscale organization of clinically relevant dynamics.

## 4 Results

To visualize dynamic spatial network variations prior to spectral decomposition, Figure 2 displays the original broadband NeuroMark network maps for two representative components (Network 6 and Network 49) from Subject 100 across four temporal windows (w = 10, 20, 30, 40). Although the overall canonical topology remains stable across time, window‐to‐window intensity differences are clearly observable, indicating fluctuations in spatial network expression within the dynamic fMRI signal. These variations represent the intrinsic spatial dynamics captured in Stage A of our pipeline.

**FIGURE 2:**
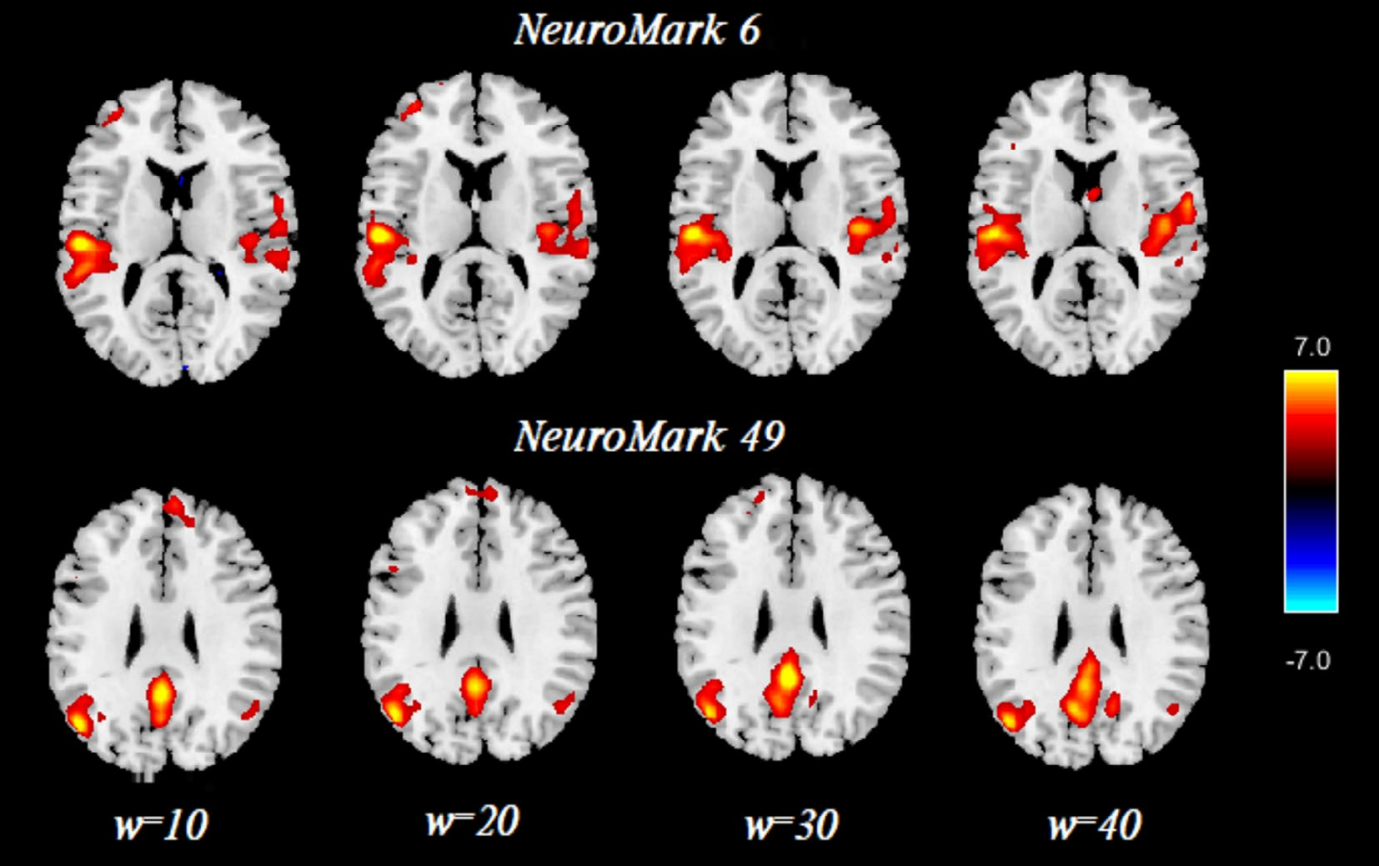
Dynamic spatial network maps before spatial frequency decomposition. Example spatial maps of NeuroMark components #6 and #49 from Subject 100 at four temporal windows (w = 10, 20, 30, 40). Each map represents the broadband spatial pattern estimated using NeuroMark‐DyFICA in Stage A (Figure 1A). These results illustrate that the canonical spatial layout of intrinsic networks is preserved across time, while subtle changes in spatial intensity patterns emerge across windows, reflecting time‐varying fluctuations in network expression.

To further investigate whether dynamic variability differs across spatial scales, we decomposed each dynamic spatial map into four spatial‐frequency bands. Figure 3 presents the resulting frequency‐resolved spatial maps for the same two networks shown in Figure 2 (NeuroMark 6 and NeuroMark 49) at representative windows 10 and 30, illustrating how fine‐ and coarse‐scale spatial features evolve over time within each network. Lower spatial‐frequency bands preserve broader and smoother spatial organization, whereas higher‐frequency bands emphasize more localized and heterogeneous spatial features that are less prominent in the broadband maps. In contrast, higher‐frequency bands emphasize finer spatial features, including localized hotspots and distributed sub‐regions that are less prominent in the broadband maps. Notably, the spatial distribution of high‐frequency content differs between windows, suggesting frequency‐specific spatial modulation across time.

**FIGURE 3:**
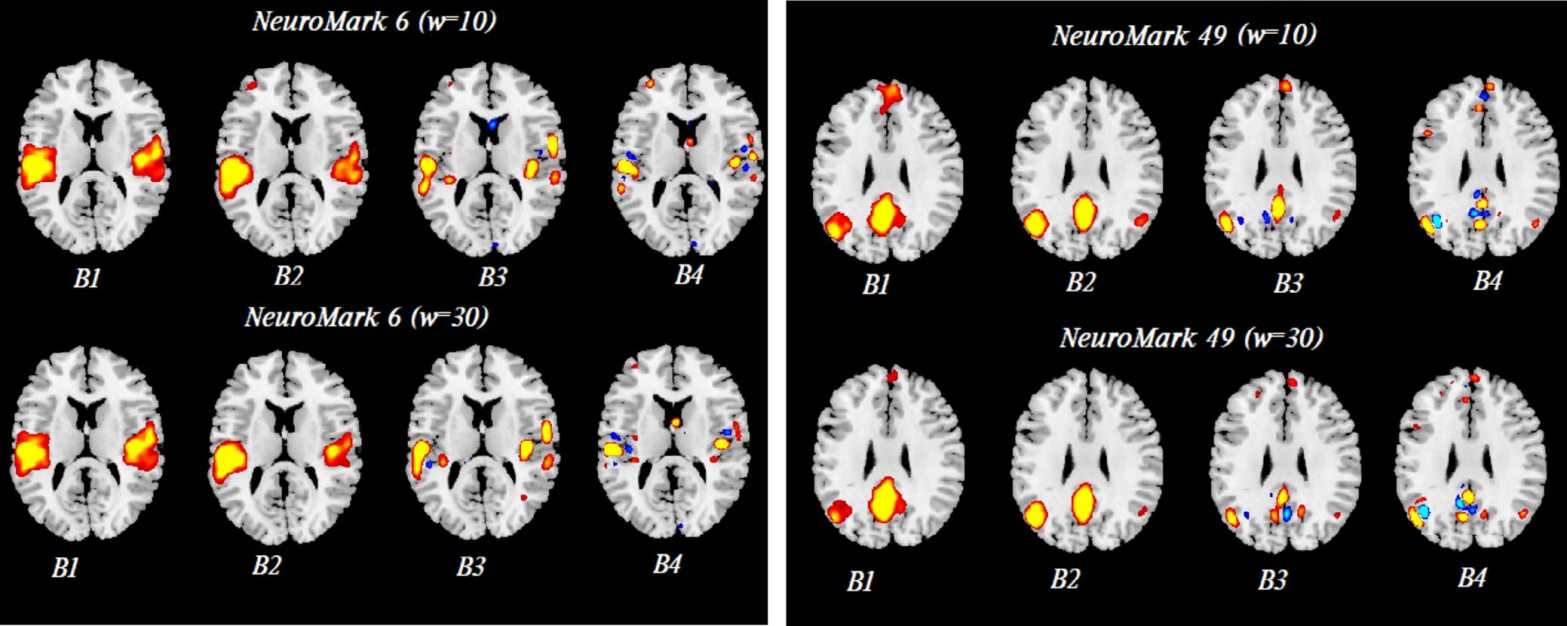
Spatial‐frequency–resolved maps obtained using four‐band spatial filtering. Spatial maps of NeuroMark components *#6* and *#49* for Subject 100 after spatial band‐pass decomposition (Stage B in Figure *1*B). For each network, maps are shown for four frequency bands ([0.01–0.0625], [0.0625–0.125], [0.125–0.1875], [0.19–0.25]) *at* two temporal windows (*w* = 10 and *w* = 30). Lower frequency bands display smooth, large‐scale network structure, while higher‐frequency bands highlight more localized, fine‐grained spatial modulations that are not apparent in broadband maps. A display threshold of 3 was applied (this threshold was selected to highlight the most salient spatial patterns while suppressing background noise), and the color bar represents the Z‐score.

Group‐level ICA identified six frequency‐resolved components for each analyzed NeuroMark network. Figure 4 illustrates the resulting multiscale spatial organization for a representative subject. Across the four spatial‐frequency bands, the components exhibited progressively finer spatial features, with lower‐frequency representations retaining broader network organization and higher‐frequency representations showing more localized spatial structure.

**FIGURE 4:**
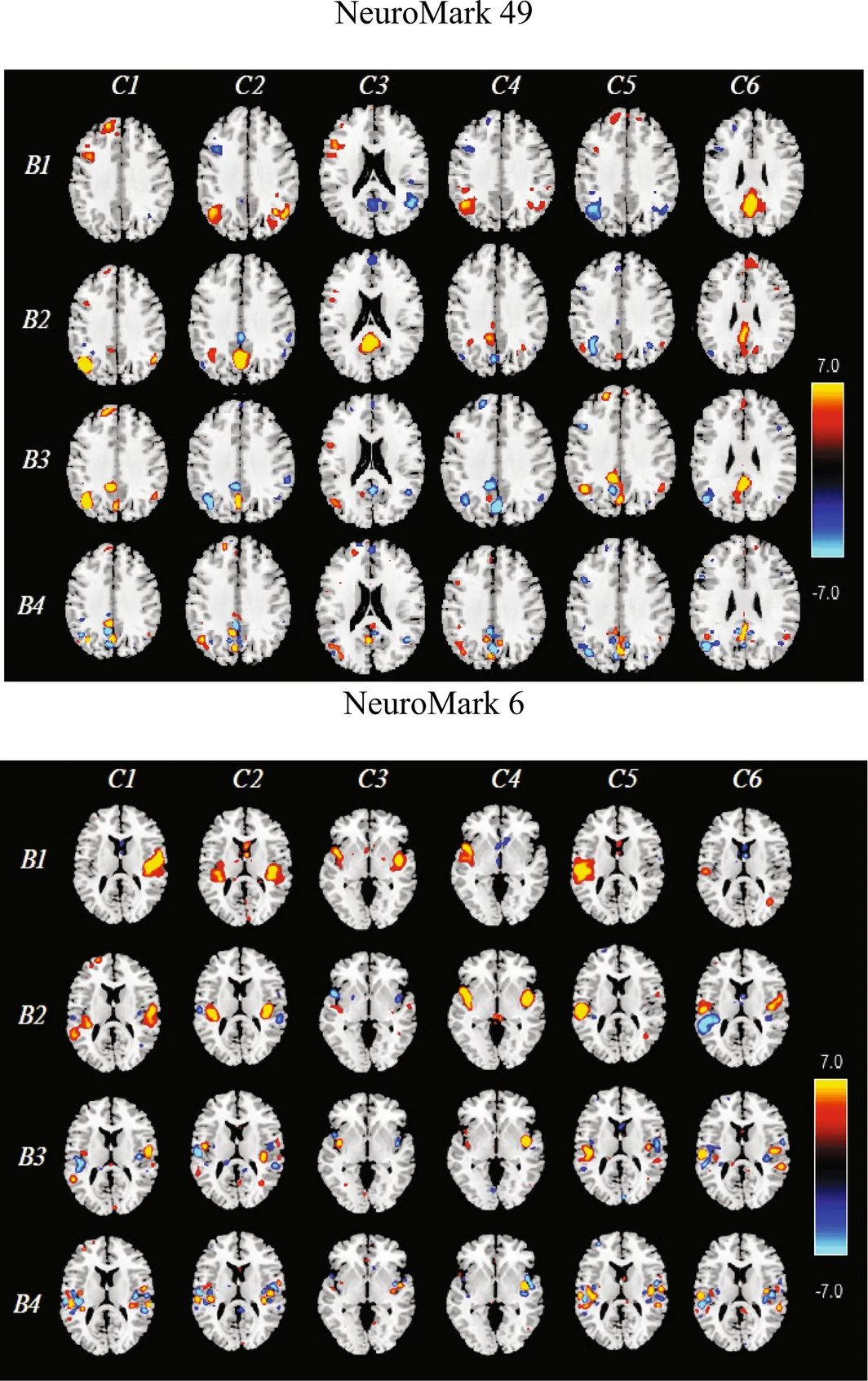
Group‐level multiscale components of NeuroMark components *#49* and *#6* for Subject 100 extracted via blind ICA on the concatenated window‐band dataset (Stage C). Each component is originally estimated as a $\left(4x\right)\times y\times z$ volume, then partitioned to four X × Y × Z maps representing individual spatial frequency bands. Low bands reflect coarse topography, while high bands reveal fine‐scale substructure. These components serve as reference maps for Stage D constrained ICA. A display threshold of 3 was applied (this threshold was selected to highlight the most salient spatial patterns while suppressing background noise), and the color bar represents the Z‐score.

To evaluate the reproducibility of the group‐level frequency‐resolved ICA decomposition, we applied the ICASSO framework across repeated ICA runs with different random initializations. The resulting components showed high stability across runs, with Iq values exceeding 0.9 for the extracted components. These results indicate that the identified latent spatial‐frequency components were highly consistent across independent ICA initializations, supporting the reproducibility and stability of the group‐level decomposition.

Subject‐level reconstruction produced frequency‐resolved spatial representations for the six NeuroMark networks of interest (Networks 3, 6, 22, 36, 49, and 52). Figure 5 presents the corresponding group‐mean spatial maps across the four spatial‐frequency bands.

**FIGURE 5:**
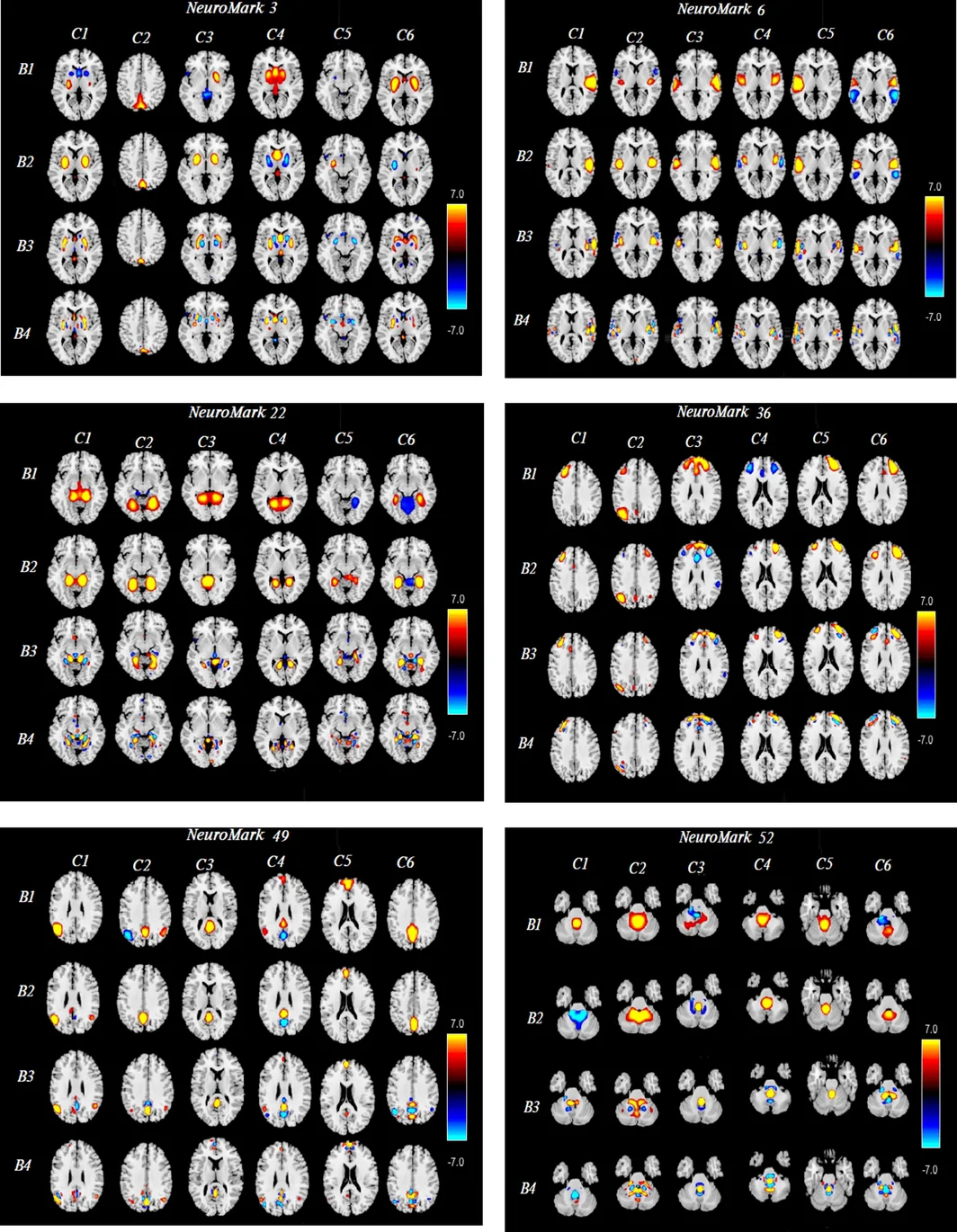
Group‐mean spatial maps of six NeuroMark networks (3, 6, 22, 36, 49, and 52) extracted using spatially constrained ICA (Figure 1. Stage D). Rows represent the six ICA components associated with each network, and columns correspond to the four frequency bands. Spatially constrained ICA was performed separately for each subject and each band, and the displayed maps reflect the mean component patterns across subjects. The results demonstrate that NeuroMark‐guided decomposition preserves network correspondence across participants while expressing frequency‐specific spatial variability. A display threshold of 3 was applied, and the color bar represents the Z‐score.

The maps preserved the canonical organization of the NeuroMark networks while showing frequency‐dependent spatial variations. Lower‐frequency components retained broader and smoother network organization, whereas higher‐frequency components exhibited more localized spatial features. Some spatial motifs were observed across multiple frequency bands, indicating multiscale organization within the same functional networks rather than enforced correspondence of component indices across bands.

To investigate interaction patterns among frequency‐resolved ICA components, we quantified both long‐term and short‐term dynamic correlations across subjects. For each network, the windowed feature matrices from the selected NeuroMark networks (49 windows × 6 components × 4 frequency bands) were concatenated, forming a subject‐level representation with dimensions *49 ×* (*6 components × 4 bands × number of networks*). The full correlation matrix was computed for each subject, capturing long‐term association strength across components and networks. In parallel, to estimate temporal variability of connectivity, a short‐term measure was calculated by segmenting the window series into non‐overlapping blocks of six windows (≈short‐term windows). Correlation matrices were computed within each segment, and the standard deviation of correlations across segments was used as short‐term variability. Group differences between patients and controls were assessed using element‐wise two‐sample *t*‐tests followed by a *single global FDR correction* applied only once over the lower triangle of each matrix, ensuring conservative control of false discoveries across all network pairs. For interpretability, results were visualized as signed statistical maps ($-\log10\left(p\right)\times \mathrm{sign}\left(t\right)$) where positive values indicate stronger connectivity/variability in patients, and negative values indicate higher values in controls. Network blocks and within‐network component boundaries were annotated in the matrix structure to facilitate interpretation of intra‐ and inter‐network contributions. Figures 6 and 7 depict the group‐level results for long‐term correlation and short‐term variability, respectively. White asterisks mark connections surviving FDR < 0.05. These visualizations allow identification of altered connectivity patterns within and across networks as well as band‐specific component interactions. Furthermore, inset zoom panels demonstrate a focused view on one selected block for clearer representation of significant links.

**FIGURE 6:**
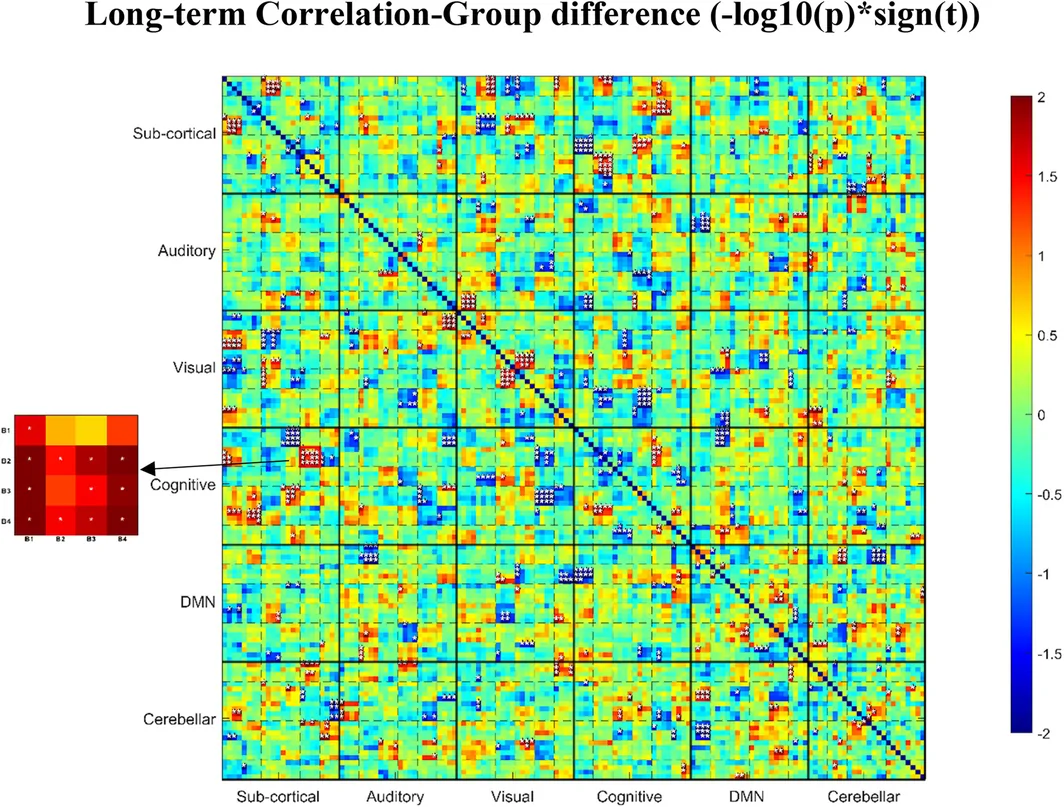
Group‐level differences in long‐term correlation across frequency‐resolved ICA components. The matrix *displays* signed statistical values ($-\log10\left(p\right)\times \text{signt}$) comparing SZ versus HC. Rows/columns are grouped by networks (sub‐cortical, auditory, visual, cognitive control, default mode, cerebellar), and component boundaries appear inside each network block. Warmer colors indicate higher connectivity in SZ, while cooler colors represent stronger connectivity in HC. White asterisks mark FDR‐significant connections (global FDR < 0.05). The inset panel highlights a selected network/component block for detailed visualization (Long‐term functional correlation between component 2 of the cognitive network and component 5 of the subcortical network). The color bar represents the significance strength.

**FIGURE 7:**
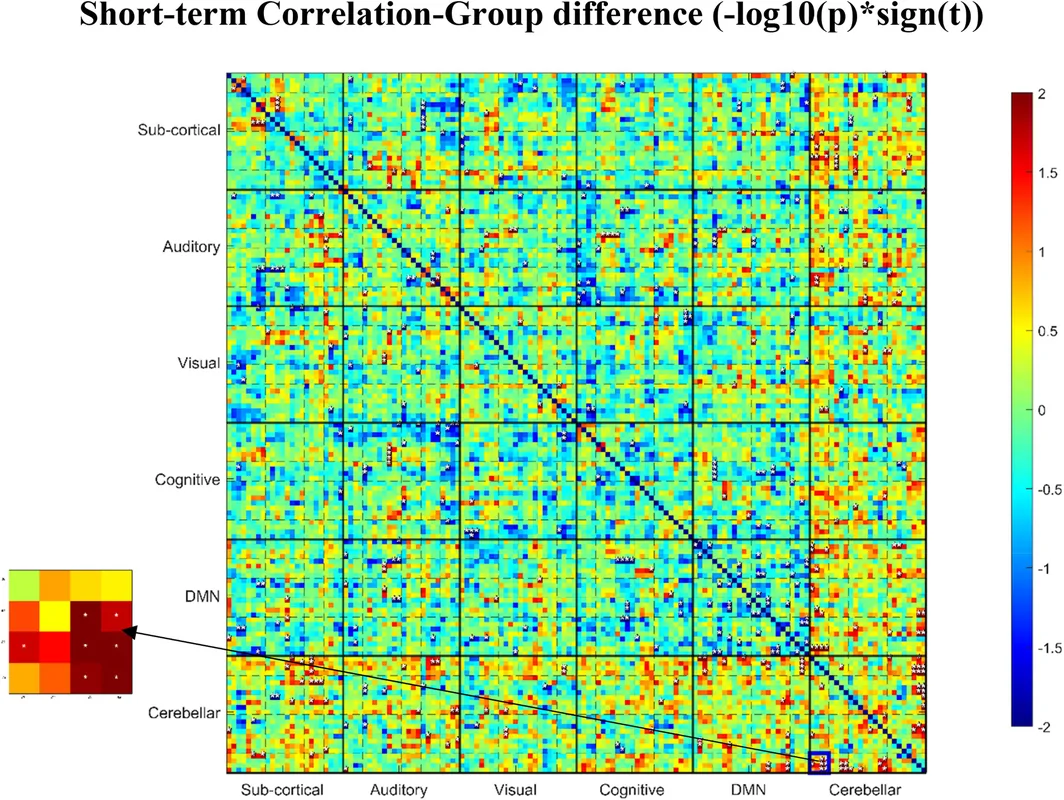
Group‐level differences in short‐term correlation variability (temporal instability). Same visualization structure as Figure 6, but each cell represents the standard deviation of the segment‐wise correlation values across short‐term segments, reflecting dynamic fluctuations rather than static connectivity. Positive values indicate greater temporal variability in patients, and negative values reflect more stable connectivity in controls. FDR‐significant values are marked with white asterisks. Network and component boundaries are shown for interpretability, with an inset zoom panel for a selected component block (short‐term correlation variability between component 6 and 1 of the cerebellar network). The color bar represents the significance strength.

After computing group‐level statistical matrices for long‐term and short‐term dynamic connectivity, we visualized significant correlations within each network separately, allowing a clearer representation of component‐ and frequency‐specific effects. For each NeuroMark network, only connections that survived the *global FDR correction* (*q < 0.05*) in the long‐term or short term correlation analysis were retained. Throughout the Results, each ICA component is identified by its NeuroMark network and component index (e.g., NM49‐C1 denotes component 1 of NeuroMark‐49), ensuring that component identities remain unambiguous across networks. To improve interpretability, we represented all frequency‐resolved component maps as thumbnails arranged on a circular/elliptical layout, with nodes labeled according to the corresponding NeuroMark network and component index (e.g., NM49‐C1), and grouped and color‐coded by frequency band (B1–B4). Edges were drawn only between significant component pairs, and line color reflected the statistical direction and effect strength using ($-\log10\left(p\right)\times \text{signt}$). Warm colors indicate stronger connectivity in SZ, whereas cool colors denote stronger connectivity in HC; edge thickness reflects the magnitude of the corresponding group difference (Figures 8, 9, 10). This visualization clearly indicates how dynamic spectral components of each network contribute to long‐term/short‐term correlation alterations across groups. Importantly, this view allows us to identify *which band(s) and which component(s)* drive the observed group separation in Figures 6/7 (full‐matrix results).

**FIGURE 8:**
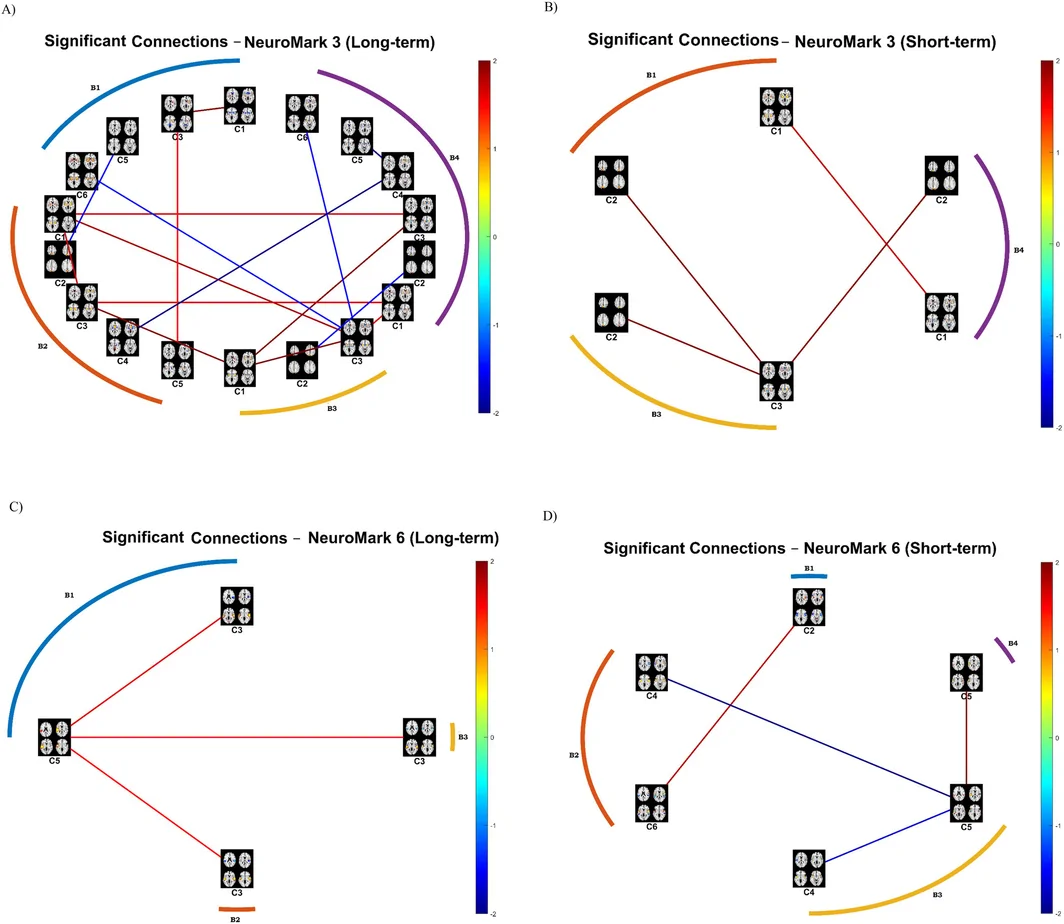
Significant (A, C) long‐term and (B, D) short‐term dynamic connectivity links in the sub‐cortical (NeuroMark‐3) and auditory (NeuroMark‐6) network. Circular visualization of FDR‐significant correlations (q<0.05) among frequency‐resolved ICA components. Each thumbnail represents a network‐specific ICA component identified by the corresponding NeuroMark network and component index (e.g., NM6‐C1), across the four spatial‐frequency bands (B1–B4). Component indices are therefore interpreted only within their corresponding NeuroMark network. Edges indicate component‐to‐component connectivity differences between groups, where edge color reflects $-\log10\left(p\right)\times \mathrm{sign}\left(t\right)$ (warm/red = SZ>HC, cool/blue = HC>SZ); color intensity represents significance magnitude. Only connections that survived global FDR correction are shown. Colored arcs denote band‐wise grouping, enabling interpretation of frequency‐specific alterations. The color bar illustrates values of $-\log10\left(p\right)\times \mathrm{sign}\left(t\right)$ (positive = SZ‐enhanced connectivity; negative = HC‐enhanced connectivity).

**FIGURE 9:**
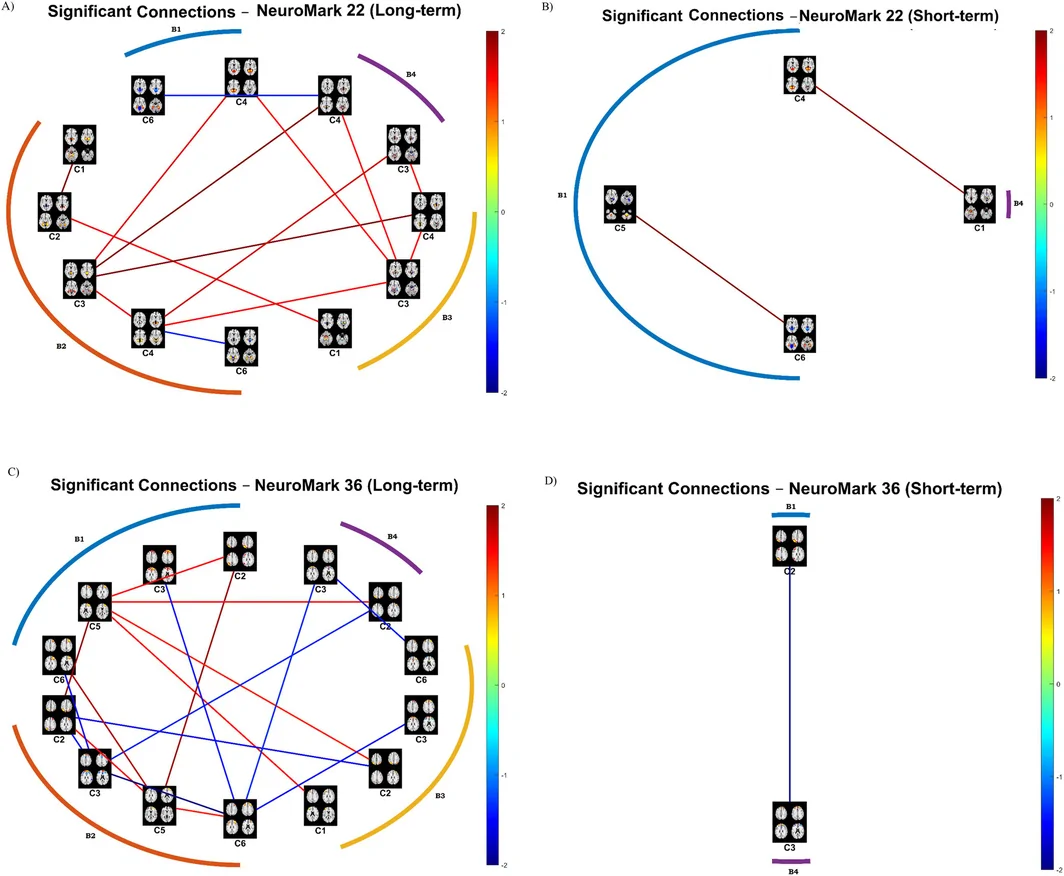
Significant (A, C) long‐term and (B, D) short‐term dynamic connectivity links in the visual (NeuroMark‐22) and cognitive (NeuroMark‐36) network. Circular visualization of FDR‐significant correlations (q<0.05) among frequency‐resolved ICA components. Each thumbnail represents a network‐specific ICA component identified by the corresponding NeuroMark network and component index (e.g., NM6‐C1), across the four spatial‐frequency bands (B1–B4). Component indices are therefore interpreted only within their corresponding NeuroMark network. Edges indicate component‐to‐component connectivity differences between groups, where edge color reflects $-\log10\left(p\right)\times \mathrm{sign}\left(t\right)$ (warm/red = SZ>HC, cool/blue = HC>SZ); color intensity represents significance magnitude. Only connections that survived global FDR correction are shown. Colored arcs denote band‐wise grouping, enabling interpretation of frequency‐specific alterations. The color bar illustrates values of $-\log10\left(p\right)\times \mathrm{sign}\left(t\right)$ (positive = SZ‐enhanced connectivity; negative = HC‐enhanced connectivity).

**FIGURE 10:**
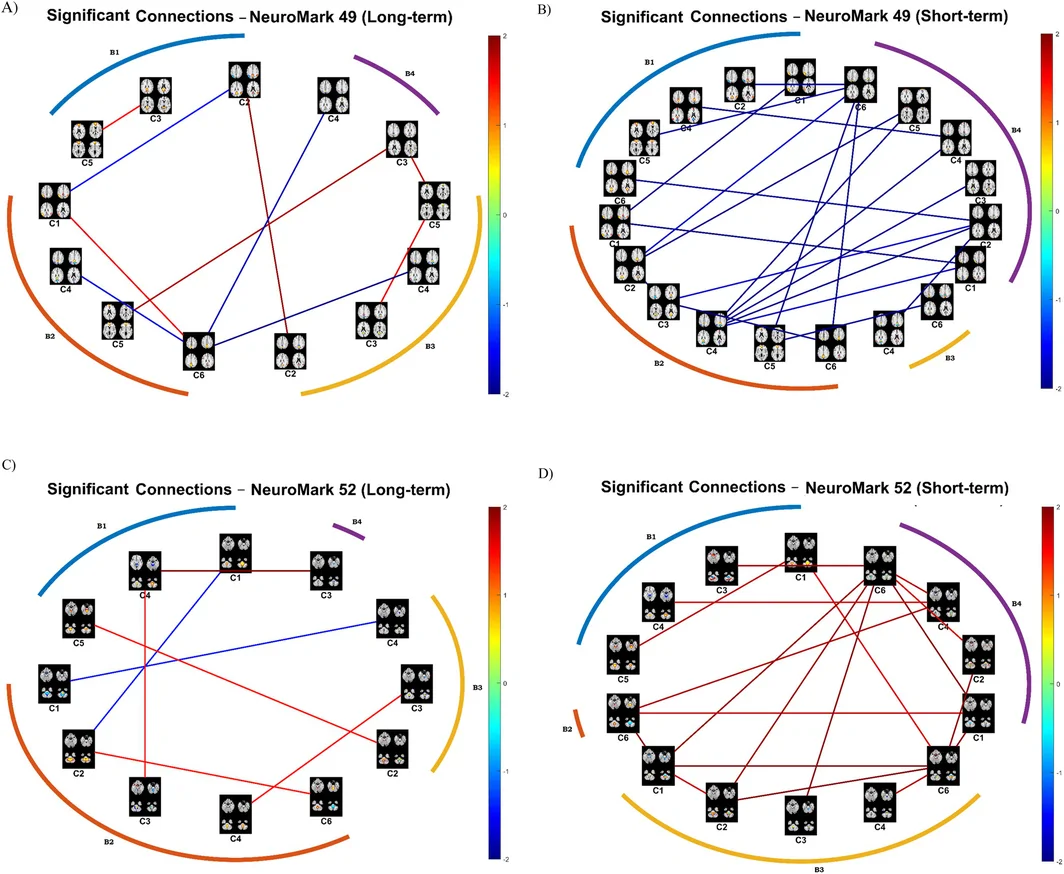
Significant (A, C) long‐term and (B, D) short‐term dynamic connectivity links in the DMN (NeuroMark‐49) and cerebellar (NeuroMark‐52) network. Circular visualization of FDR‐significant correlations (q<0.05) among frequency‐resolved ICA components. Each thumbnail represents a network‐specific ICA component identified by the corresponding NeuroMark network and component index (e.g., NM6‐C1), across the four spatial‐frequency bands (B1–B4). Component indices are therefore interpreted only within their corresponding NeuroMark network. Edges indicate component‐to‐component connectivity differences between groups, where edge color reflects $-\log10\left(p\right)\times \mathrm{sign}\left(t\right)$ (warm/red = SZ>HC, cool/blue = HC>SZ); color intensity represents significance magnitude. Only connections that survived global FDR correction are shown. Colored arcs denote band‐wise grouping, enabling interpretation of frequency‐specific alterations. The color bar illustrates values of $-\log10\left(p\right)\times \mathrm{sign}\left(t\right)$ (positive = SZ‐enhanced connectivity; negative = HC‐enhanced connectivity).

Long‐term connectivity patterns revealed stable and structured alterations, with more persistent positive (SZ‐enhanced) interactions across multiple NeuroMark templates. In NeuroMark‐3, long‐term correlations were dominated by red connections linking NM3‐C1–NM3‐C3 components across multiple bands, suggesting stronger long‐term cross‐component coupling in SZ. Similar SZ‐dominant long‐range coupling differences were observed across NeuroMark‐22, NeuroMark‐36, NeuroMark‐49, and NeuroMark‐52. These alterations involved several network‐specific component pairs, including C3–C4, C3–C5, and C2–C5/C6 across spatial‐frequency bands. A smaller number of blue (HC‐enhanced) connections appeared selectively (e.g., NM22‐C4‐B2–NM22‐C6‐B2 in NeuroMark‐22 and NM36‐C3‐B4–NM36‐C6‐B2 in NeuroMark‐36), indicating higher correlation values in HC than SZ for these connections. Overall, long‐term dynamics revealed stronger positive coupling among several components in SZ, contrasted with stronger negative or weaker positive coupling in selected connections in HC, suggesting altered long‐range network coordination in SZ. Short‐term connectivity displayed a more fragmented and frequency‐dependent pattern, suggesting transient fluctuations rather than sustained coupling. In NeuroMark‐52, red SZ‐dominant connections were highly concentrated around NM52‐C1:NM52‐C6 and NM52‐C1:NM52‐C5 pairs across multiple bands, along with dense NM52‐C6 cross‐band interactions (B3/B4). Greater short‐term correlation variability was also observed in NeuroMark‐6 (NM6‐C5‐B3:NM6‐C5‐B4, NM6‐C6‐B2:NM6‐C2‐B1) and NeuroMark‐3 (NM3‐C1‐B1:NM3‐C1‐B4 and cross‐frequency interactions involving NM3‐C2 and NM3‐C3), suggesting greater temporal fluctuations in cross‐frequency coupling in SZ. In contrast, healthy‐enhanced blue links were more numerous in NeuroMark 49, involving interactions among NM49‐C6, NM49‐C5, NM49‐C4, and NM49‐C2 across multiple bands, with fewer HC‐enhanced connections observed in NeuroMark‐36 and NeuroMark‐6. This pattern indicates greater short‐term fluctuations in cross‐band coupling in SZ, whereas HC showed higher correlation values in several connections.

Combining the long‐ and short‐term findings suggests that SZ connectivity abnormalities may involve two complementary patterns: (1) greater short‐term variability in cross‐frequency coupling, with several effects involving components such as NM52‐C1, NM52‐C5, NM52‐C6, and corresponding network‐specific components in other NeuroMark networks, and (2) stronger long‐term positive coupling in selected component pairs, including NM49‐C3–NM49‐C4 and NM49‐C3–NM49‐C5, as well as corresponding network‐specific interactions in other networks. Conversely, HC showed higher correlation values in several short‐term connections, indicating relatively greater temporal stability or different coupling patterns in these component pairs. Together, these findings suggest that SZ is associated with both greater short‐term fluctuations in cross‐frequency coupling and altered long‐term coupling among selected component pairs, indicating differences in temporal coordination and network organization across spatial and temporal scales To assess the behavioral and clinical relevance of the proposed framework, we examined associations between frequency‐resolved dynamic connectivity metrics and cognitive and symptom measures within the SZ group. Across analyses, both long‐term and short‐term dynamic metrics exhibited significant associations with speed of processing, working memory, and PANSS symptom severity after FDR correction, suggesting that multiscale connectivity dynamics are associated with clinically meaningful variability. Overall, long‐term dynamics showed a greater number of significant associations with working memory and PANSS negative symptoms, whereas short‐term dynamics were more strongly associated with speed of processing. PANSS positive symptoms demonstrated an intermediate pattern, with fewer significant associations compared to PANSS‐N but greater involvement in long‐term than short‐term dynamics.

Examination of spatial frequency contributions revealed a clear dissociation across temporal scales (Figure 11). Long‐term clinical associations were more frequently observed in lower spatial frequency bands whereas short‐term associations exhibited relatively greater contributions from higher spatial‐frequency bands, particularly for speed of processing. This pattern suggests that broader spatial organization may contribute more strongly to long‐term clinical associations, whereas finer‐scale spatial features may be more relevant to short‐term associations.

**FIGURE 11:**
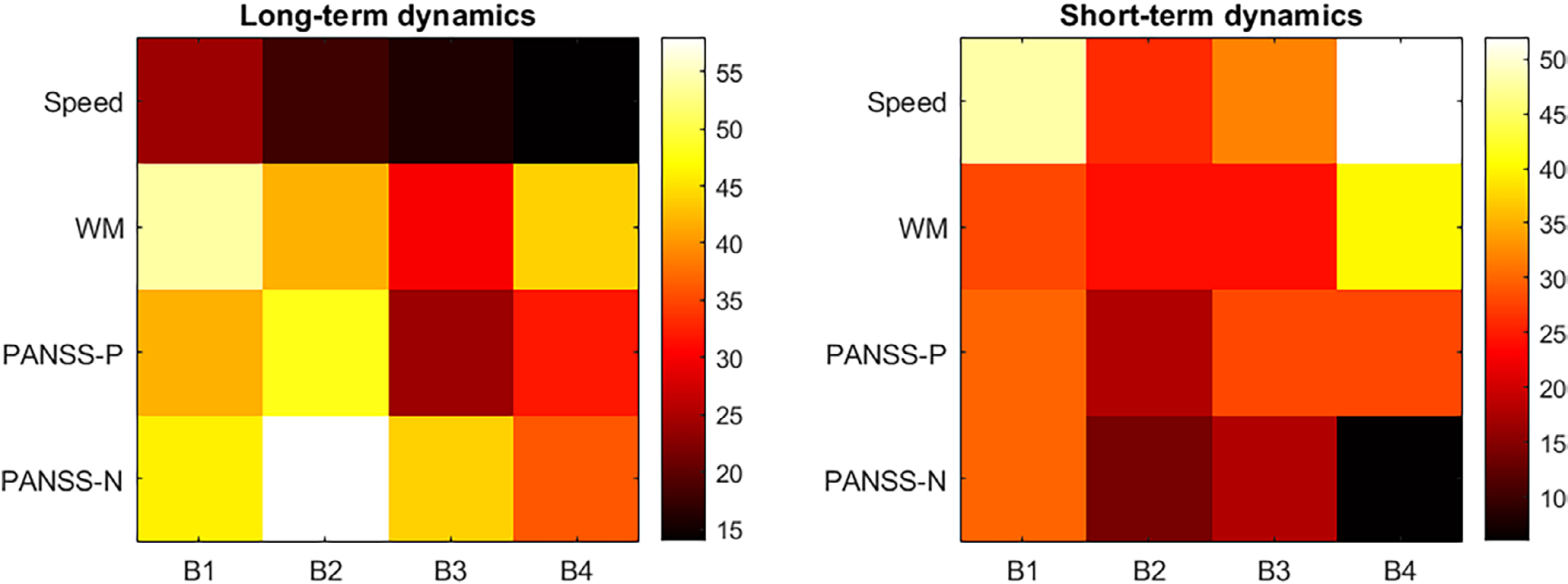
Spatial‐frequency–specific associations between dynamic connectivity and clinical measures in SZ. Distribution of FDR‐significant Spearman correlations across spatial frequency bands for long‐term and short‐term dynamic connectivity metrics, evaluated against speed of processing, working memory, and PANSS symptom scores.

Component‐wise analysis indicated that clinically relevant associations were driven by a subset of components rather than being uniformly distributed across all components (Figure 12), highlighting the presence of specific dynamic subsystems linked to cognitive and symptom variability. At the network level, significant associations were concentrated within higher‐order cognitive, default‐mode, and cerebellar networks (Figure 13), with long‐term dynamics showing broader network involvement and short‐term dynamics exhibiting more focal patterns.

**FIGURE 12:**
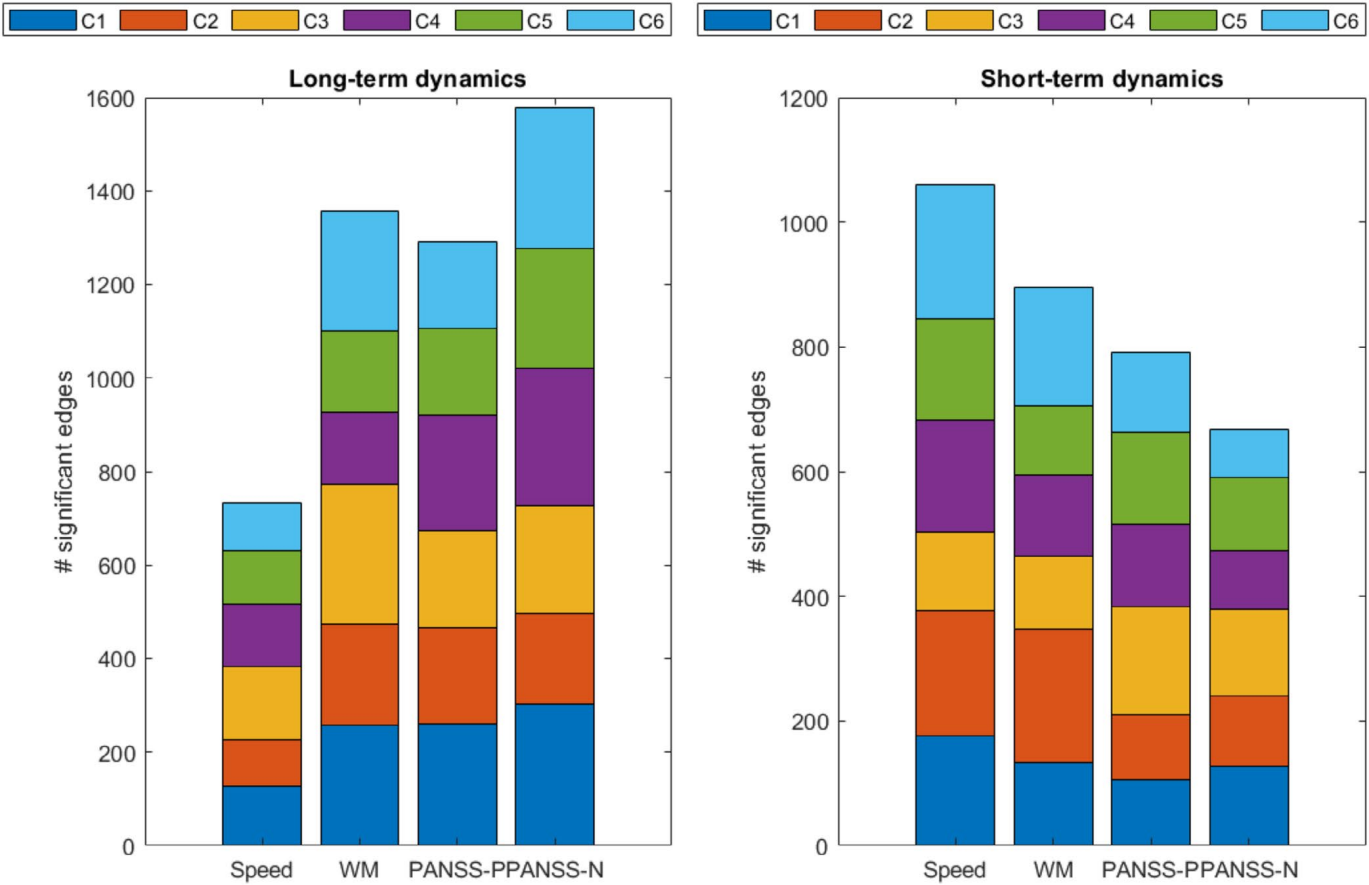
Component‐level contribution to clinical associations across temporal scales. Relative contribution of ICA components to FDR‐significant associations with clinical and cognitive measures, shown separately for long‐term and short‐term dynamics.

**FIGURE 13:**
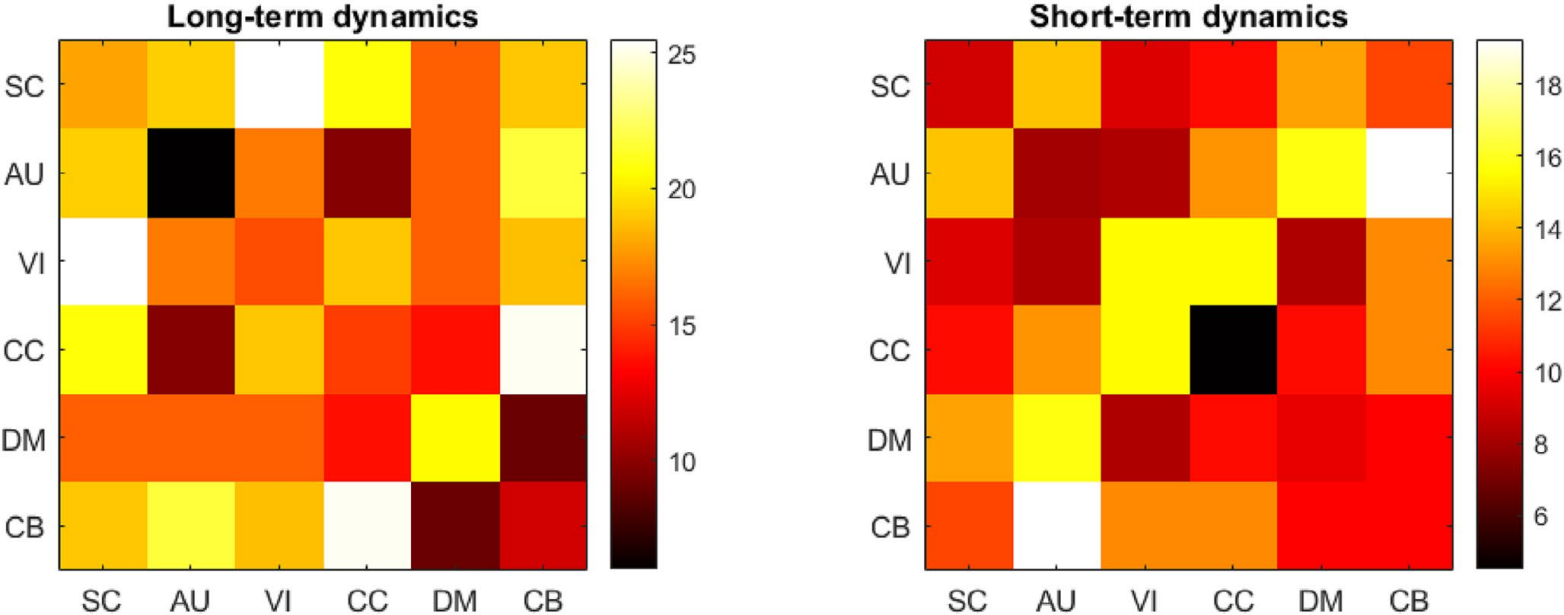
Network‐level distribution of clinically relevant dynamic connectivity patterns. Proportion of significant associations across NeuroMark functional networks, highlighting distinct network involvement for long‐term versus short‐term dynamic connectivity.

## 5 Discussion

In this work, we introduced SPARK–ICA, a frequency‐resolved dynamic spatial ICA framework that extends existing dynamic fMRI approaches by explicitly modeling spatial‐frequency–specific network reconfigurations over time. Unlike conventional dynamic functional connectivity methods that primarily focus on temporal fluctuations between fixed spatial patterns (Preti et al. 2017; Allen et al. 2014), our approach reveals how fine‐ and coarse‐scale spatial features within canonical brain networks dynamically reorganize and how these reorganizations differ between SZ and HC.

An important distinction from our previous NeuroMark‐DyFICA framework (Behzadfar et al. 2026) is that SPARK‐ICA does not simply provide another estimate of dynamic spatial states. NeuroMark‐DyFICA was designed to identify transient fine‐scale spatial reconfigurations by combining template‐guided dynamic ICA with high‐pass spatial filtering and subsequent group‐level decomposition. In contrast, SPARK‐ICA explicitly resolves the spatial organization of these dynamics across multiple spatial‐frequency bands. Thus, the two approaches address related but distinct aspects of dynamic network organization: NeuroMark‐DyFICA emphasizes dynamic deviations from canonical network topography, whereas SPARK‐ICA further characterizes how such network organization is distributed across spatial scales and how these spatial scales interact with different temporal regimes.

Most dynamic ICA and dFNC studies assume that network spatial maps are static, allowing only their temporal expression or inter‐network coupling to vary (Allen et al. 2014; Calhoun et al. 2001). However, growing evidence suggests that network topography itself exhibits meaningful temporal variability, even within canonical resting‐state networks (Iraji, Fu, et al. 2019; Miller et al. 2016). By decomposing dynamic spatial maps into multiple spatial‐frequency bands, SPARK–ICA explicitly captures scale‐dependent spatial dynamics, enabling the separation of large‐scale smooth organization from finer subregional modulations that are otherwise conflated in broadband analyses.

The use of NeuroMark template guidance promotes reproducibility and cross‐subject correspondence, addressing a known limitation of purely blind dynamic ICA methods (Calhoun and Adali 2012; Du 2020). In addition, the ICASSO analysis provides an empirical assessment of the stability of the group‐level ICA decomposition. Importantly, the group‐level frequency‐resolved ICA step provides a latent spatial‐frequency reference space, which allows subject‐ and band‐specific dynamics to be estimated while preserving interpretability across individuals and groups. Once learned, this reference space can be directly reused to analyze new, previously unseen subjects via spatially constrained ICA, enabling scalable extension of the framework without re‐estimating the group model. This combination of template guidance and frequency‐resolved modeling builds directly on prior spatially constrained dynamic ICA work (Iraji, Fu, et al. 2019) while extending it into the spatial‐frequency domain.

Notably, while components are estimated independently within each spatial‐frequency band, partial spatial correspondence across bands was observed for a limited number of components, suggesting that certain network motifs may be expressed across multiple spatial scales. Rather than reflecting duplicated components, such multi‐scale correspondence likely indicates shared underlying organization that manifests differently depending on spatial‐frequency content. This multi‐band representation provides an aspect of network organization that is not available from a broadband dynamic ICA representation alone. Whereas a broadband analysis combines spatial features across scales into a single spatial map, SPARK‐ICA separates these contributions and allows their temporal dynamics to be examined independently. The purpose of this decomposition is therefore not to imply that abnormalities identified in broadband analyses are absent, but to determine whether their expression is preferentially associated with particular spatial scales.

Our long‐term correlation analysis reveals network‐specific and frequency‐dependent alterations rather than a uniform pattern of dysconnectivity in SZ. Within the visual network (NeuroMark 22), SZ subjects exhibit predominantly stronger long‐term correlations across multiple component–band pairs, suggesting abnormally stable integration of visual network subcomponents, particularly involving higher‐frequency spatial features. This finding aligns with prior reports of visual system hyperconnectivity and altered sensory processing in SZ (Anticevic et al. 2014; Javitt 2009), and extends them by showing that these abnormalities are preferentially expressed at specific spatial scales.

These findings can also be considered in relation to our previous NeuroMark‐DyFICA analysis of the same fBIRN dataset (Behzadfar et al. 2026). NeuroMark‐DyFICA identified schizophrenia‐related alterations in the distribution and convergence of dynamic spatial states, including abnormalities in the visual/fusiform, middle frontal, DMN, auditory, and cerebellar systems. SPARK‐ICA identifies related abnormalities in several of these systems, including visual, subcortical, cognitive‐control, DMN, and cerebellar networks, but further resolves their expression according to spatial‐frequency band and temporal scale. In particular, the distinction between long‐term and short‐term correlations, together with the differential involvement of lower‐ and higher‐frequency spatial components, provides information about the spatial scale at which dynamic abnormalities are expressed. Thus, the present findings should be viewed as complementary to, rather than a replacement for, the dynamic state abnormalities identified by NeuroMark‐DyFICA.

In contrast, the sub‐cortical (NeuroMark 3) and cognitive‐control (NeuroMark 36) networks show more HC‐dominant long‐term connectivity, indicating stronger stable coordination of subcomponents in HCs. Given the central role of subcortical–cortical interactions in dopaminergic regulation and cognitive control (Howes and Kapur 2009; Cole et al. 2014) reduced long‐term integration in SZ may reflect impaired stabilization of these circuits over extended timescales. Notably, these effects are not restricted to a single frequency band but involve cross‐band interactions, underscoring the importance of multi‐scale spatial analysis.

The DMN (NeuroMark 49) exhibits a mixed pattern of SZ‐ and HC‐dominant long‐term connections, consistent with prior reports of heterogeneous DMN alterations in SZ (Whitfield‐Gabrieli and Ford 2012). Our results further suggest that this heterogeneity is scale‐dependent, with different spatial‐frequency components contributing differently to group separation.

Short‐term correlation analysis reveals a complementary pattern of dysfunction in SZ, characterized by pronounced alterations in rapid, high‐frequency interactions. Notably, the cerebellar network (NeuroMark 52) emerges as a particularly prominent locus of abnormality, exhibiting widespread short‐term hyperconnectivity with multiple other networks rather than isolated pairwise effects.

Interpretation of higher spatial‐frequency components warrants some caution, as these components may be more sensitive to partial‐volume effects and CSF‐adjacent regions. Nevertheless, the observed group differences were distributed across multiple components and networks rather than being confined to isolated components, increasing confidence that these findings are not attributable to a single spatial feature.

The broad involvement of the cerebellar network suggests that cerebellar dynamics may represent an important component of short‐timescale dysregulation in SZ, consistent with the cerebellum's proposed function as a temporal coordinator and modulator of distributed brain activity. In parallel, significant short‐term alterations are also observed within the subcortical network, further implicating fast subcortical–cortical interactions in SZ. These findings are consistent with models of SZ that emphasize increased neural variability and reduced temporal coordination at fast timescales, particularly affecting cerebellar and subcortical systems involved in timing, prediction, and sensorimotor integration (Anticevic et al. 2014; Uhlhaas and Singer 2010). Importantly, the preferential involvement of higher spatial‐frequency components indicates that short‐term abnormalities are associated with fine‐grained spatial instabilities and rapidly fluctuating local network configurations, providing a spatial‐frequency‐resolved perspective that complements approaches focused primarily on large‐scale topological reorganization of functional networks.

Importantly, higher‐order networks such as cognitive‐control (NeuroMark 36) show fewer SZ‐dominant short‐term connections, suggesting that deficits in these systems may be more closely related to impairments in long‐term integration rather than transient fluctuations. This dissociation between long‐term and short‐term alterations highlights the Supporting Information provided by explicitly modeling spatial‐frequency–resolved dynamics.

In addition to group‐level differences, the observed associations between frequency‐resolved dynamic connectivity and cognitive and clinical measures further support the functional relevance of the proposed framework. Importantly, these relationships were not uniform across temporal scales or spatial frequency bands, indicating that distinct aspects of network dynamics contribute to different behavioral and symptom dimensions in SZ. Long‐term dynamic connectivity exhibited stronger associations with WM performance and negative symptom severity, suggesting that stable, lower‐frequency spatial organization may be particularly relevant to sustained cognitive integration and motivational processes in SZ. Alterations in these slower, more persistent connectivity patterns may therefore reflect impaired maintenance of coordinated network activity, a hallmark of negative symptoms and higher‐order cognitive deficits in SZ.

In contrast, short‐term dynamic connectivity showed relatively stronger associations with speed of processing, suggesting that rapid fluctuations and fine‐scale spatial reconfigurations may be particularly relevant for fast perceptual–motor and attentional operations. The spatial frequency–specific nature of these findings provides insight beyond conventional dynamic connectivity analyses. Lower spatial frequency bands primarily contributed to long‐term clinical associations, whereas higher spatial frequencies were more prominently involved in short‐term associations, particularly for processing speed. This dissociation suggests that SZ‐related dysfunction may differentially affect coarse, stable network organization and fine‐grained, rapidly evolving spatial patterns.

Notably, this multiscale disruption aligns with prior spatial chronnectome studies demonstrating altered within‐ and between‐network spatial dynamics in SZ and extends this work by explicitly linking spatial frequency content to temporal dynamics and clinical relevance. Together, these results highlight the importance of jointly modeling spatial frequency and temporal variability when studying brain network dynamics. By revealing clinically meaningful associations that vary across spatial and temporal scales, SPARK–ICA provides a more spatially and temporally resolved characterization of network dysfunction than analyses that do not explicitly model spatial‐frequency content.

Collectively, these findings support a view of SZ as a disorder characterized by scale‐dependent dysregulation of dynamic brain organization, in which sensory and subcortical systems exhibit excessive or abnormally stable coupling at certain spatial scales, while higher‐order control systems fail to maintain coherent long‐term integration. By introducing spatial frequency as an explicit dimension of dynamic analysis, SPARK–ICA provides a principled framework for resolving how dynamic abnormalities are distributed across spatial scales, while supporting consistent application to new subjects and datasets through reusable group‐level templates.

A formal head‐to‐head comparison between SPARK‐ICA and NeuroMark‐DyFICA was not performed in the present study, and therefore we do not claim that SPARK‐ICA detects abnormalities that NeuroMark‐DyFICA cannot detect. Rather, the comparison with our previous analysis of the same dataset is intended to clarify the complementary information provided by the two frameworks. NeuroMark‐DyFICA characterizes dynamic changes in network topography and state organization, whereas SPARK‐ICA further resolves spatial‐frequency content within dynamic network representations and examines its relationship with different temporal regimes. A systematic benchmark in which the two frameworks are evaluated under identical analysis criteria, including matched statistical testing and quantitative measures of sensitivity and reproducibility, would be valuable in future work.

Future work may extend this framework to task‐based fMRI, multimodal data (e.g., EEG–fMRI), or longitudinal designs, and investigate whether spatial‐frequency–specific dynamic markers can improve symptom stratification or treatment prediction. More broadly, incorporating spatial scale into dynamic network models may offer new insights into both healthy brain function and neuropsychiatric disease.

### 5.1 Limitation

While SPARK–ICA provides a novel framework for characterizing frequency‐resolved dynamic spatial network organization, several limitations should be considered.

First, the proposed method is primarily designed to enhance sensitivity to fine‐scale spatial variability by explicitly decomposing dynamic spatial maps into multiple spatial frequency bands. This focus provides increased resolution for characterizing subtle, localized spatial modulations across different spatial scales, although the extent to which this improves sensitivity relative to conventional dynamic ICA approaches remains to be established through direct comparative evaluation. However, as a consequence, the framework may be less sensitive to large‐scale spatial reorganizations or global network reconfigurations that unfold over broader spatial extents. Developing extensions that jointly model fine‐ and coarse‐scale spatial dynamics within a unified representation remains an important direction for future work.

Second, SPARK–ICA relies on NeuroMark‐derived spatial priors in its initial dynamic ICA stage to promote reproducibility and cross‐subject correspondence. While this template guidance is intended to promote stable component estimation, it may also bias the decomposition toward canonical network configurations derived largely from healthy populations. As a result, rare, highly individualized, or atypical spatial patterns may be underrepresented. Although subsequent stages of the pipeline—including spatial frequency decomposition and blind group‐level ICA—allow variation beyond the initial template‐defined representation, some degree of prior influence is unavoidable. Future work could explore partially adaptive or hybrid constrained–unconstrained approaches to further balance sensitivity to novel spatial organization with reproducibility.

Third, the group‐level frequency‐resolved ICA stage aggregates spatial maps across all windows, subjects, and frequency bands prior to decomposition. While this strategy enables extraction of reproducible latent components shared across scales, it implicitly assumes that dominant spatial‐frequency patterns are sufficiently consistent across time and individuals. This assumption may limit sensitivity to transient or subject‐specific spatial features that do not align well with the group‐level latent space. Subject‐adaptive or hierarchical extensions of the group ICA model could help address this limitation. Although ICASSO demonstrated high stability of the group‐level ICA decomposition across repeated initializations, this analysis does not directly assess robustness to sampling variability, preprocessing perturbations, or recovery of known spatial‐frequency patterns; simulation and split‐half or resampling analyses would provide complementary validation in future work.

Fourth, validation in the current study was limited to resting‐state fMRI data from a single clinical population (SZ). Although the observed long‐term and short‐term connectivity alterations are broadly consistent with prior literature, further evaluation on independent datasets, additional clinical populations, and task‐based paradigms will be necessary to establish generalizability. Extension of SPARK–ICA to multimodal data (e.g., EEG–fMRI) may also provide complementary validation of frequency‐specific spatial dynamics.

Fifth, as with most dynamic fMRI analyses, results may depend on parameter choices, including window length, number of frequency bands, filter characteristics, and ICA model order. In this work, we adopted window lengths and filtering settings consistent with prior dynamic ICA studies to balance temporal sensitivity and estimation stability. However, shorter windows may improve sensitivity to rapid dynamics at the expense of reliability, while longer windows provide more stable estimates but may obscure fast fluctuations. A systematic exploration of multi‐scale or adaptive windowing strategies could further enhance robustness. In particular, the relative contribution of explicit multi‐band decomposition versus a single high‐frequency representation was not directly evaluated in the present study; therefore, the incremental benefit of the multi‐band formulation should be interpreted as a methodological advantage in representation rather than as an empirically established performance gain.

The present study also did not include a formal head‐to‐head comparison between SPARK–ICA and NeuroMark‐DyFICA using matched analyses. Although SPARK–ICA extends the predominantly high‐frequency representation of NeuroMark‐DyFICA by explicitly resolving multiple spatial‐frequency bands, the degree to which this multi‐band representation improves detection sensitivity or reproducibility relative to the previous framework remains to be quantified. Such a comparison is an important direction for future work.

Finally, although spatial frequency decomposition provides a more explicit representation of fine‐scale spatial dynamics, the neurobiological interpretation of specific spatial frequency bands remains indirect. While higher‐frequency spatial components may reflect localized and heterogeneous spatial modulations, linking these features more directly to underlying cellular, laminar, or neurophysiological mechanisms will require complementary modeling and validation studies.

## 6 Conclusion and Future Work

In this work, we introduced SPARK–ICA, a frequency‐resolved dynamic spatial ICA framework designed to capture fine‐scale spatiotemporal variability in functional brain networks. By integrating NeuroMark‐guided dynamic ICA with explicit spatial frequency decomposition and blind group‐level modeling, SPARK–ICA provides a principled way to characterize how dynamic spatial network configurations evolve across both time and spatial scale. Compared with dynamic fMRI approaches that do not explicitly resolve spatial‐frequency content, the proposed framework enables systematic investigation of frequency‐specific spatial dynamics while supporting reproducibility cross‐subject reproducibility.

Applying SPARK–ICA to resting‐state fMRI data from SZ patients and HCs revealed robust frequency‐dependent alterations in dynamic network interactions. Long‐term correlation analysis demonstrated network‐specific group differences, with visual networks exhibiting predominantly SZ‐enhanced long‐term integration, whereas sub‐cortical and cognitive‐control networks showed stronger stable coordination in HCs. The DMN displayed a mixed pattern of SZ‐ and HC‐dominant effects, suggesting heterogeneous alterations across its dynamic subcomponents. Complementarily, short‐term correlation variability highlighted pronounced group differences in higher‐frequency spatial components, particularly within sub‐cortical and cerebellar networks, indicating that rapid, fine‐scale spatial fluctuations contribute substantially to transient dysconnectivity in SZ. Importantly, these findings demonstrate that the identified abnormalities are distributed across distinct spatial‐frequency and temporal scales, providing information about the scale dependence of dynamic network dysconnectivity that is not explicitly resolved when spatial‐frequency content is analyzed in aggregate.

Together, these findings support the view that SZ‐related dysconnectivity is not uniform across spatial scales or timescales, but instead emerges from scale‐specific disruptions in dynamic spatial organization. SPARK–ICA offers a flexible and reproducible framework for characterizing such scale‐specific effects and provides new avenues for linking spatially resolved network dynamics to neuropsychiatric pathology.

The present framework also extends our previous NeuroMark‐DyFICA approach, which used template‐guided dynamic ICA followed by high‐pass spatial filtering to emphasize fine‐scale spatial variations within dynamic network maps. NeuroMark‐DyFICA therefore provided a predominantly high‐frequency representation of dynamic spatial organization, whereas SPARK–ICA explicitly partitions the spatial‐frequency range into multiple bands and estimates band‐specific latent components. This distinction allows SPARK–ICA to characterize whether dynamic abnormalities are preferentially expressed at coarse, intermediate, or fine spatial scales, rather than representing high‐frequency variation as a single aggregated spatial component. The present findings extend the high‐frequency perspective of NeuroMark‐DyFICA by showing that, within the SPARK–ICA representation, SZ‐related alterations exhibit distinct relationships with temporal dynamics across multiple spatial‐frequency bands.

In addition to group‐level effects, the present findings demonstrate that frequency‐resolved dynamic spatial connectivity is meaningfully related to cognitive performance and symptom severity within the schizophrenia group. Associations with WM, processing speed, and PANSS dimensions varied across spatial frequency bands and temporal scales, indicating that different aspects of dynamic network organization contribute to distinct clinical and behavioral domains. Notably, long‐term dynamics and lower spatial frequencies were more strongly linked to WM and negative symptoms, whereas short‐term variability and higher spatial frequencies showed greater relevance for processing speed. These results suggest that clinically relevant dysfunction in SZ may be expressed through multiscale dynamic patterns rather than a single dominant mode of dysconnectivity. By revealing such scale‐dependent associations, SPARK–ICA provides a principled framework for probing how fine‐grained spatial dynamics relate to symptom expression and cognitive impairment.

Several directions can further extend and strengthen the proposed framework. First, a direct methodological comparison with NeuroMark‐DyFICA and other dynamic ICA approaches would provide an important next step for quantifying the incremental benefit of explicit spatial‐frequency resolution. In particular, future work could evaluate SPARK–ICA and NeuroMark‐DyFICA using identical data and matched preprocessing to determine which spatial‐frequency features and group‐level effects are shared and which are specifically resolved by the multi‐band formulation. Future validation should also include simulation studies with known spatial‐frequency ground truth, as well as split‐half or resampling analyses to evaluate recovery of spatial‐frequency patterns and robustness to sampling variability.

Second, future studies could develop multi‐scale or adaptive spatial‐frequency representations, allowing the frequency bands to be data‐driven rather than fixed a priori. Such extensions may better capture individual differences and condition‐specific spatial dynamics.

Third, future studies should evaluate SPARK–ICA using functional MRI data acquired or resampled at higher spatial resolution, with smaller voxel sizes (e.g., approximately 2 mm or 1.5 mm isotropic), together with preprocessing strategies appropriately matched to the available spatial resolution. Higher‐resolution data could provide greater sensitivity to fine‐scale spatial variations and help determine whether the higher‐frequency components identified by SPARK–ICA represent reproducible spatial structure rather than residual effects constrained by the current 3‐mm voxel size and 6‐mm FWHM smoothing. Such studies would provide an important test of the generalizability and biological interpretability of the B3 and B4 frequency bands and could extend the framework toward finer spatial scales.

Fourth, while the current work focused on resting‐state fMRI, SPARK–ICA is readily extendable to task‐based paradigms, where frequency‐specific spatial reconfigurations may reflect cognitive demands, learning, or adaptive control. Applying the framework to longitudinal or developmental datasets may also provide insight into how spatial‐frequency–dependent dynamics evolve over time or disease progression.

Fifth, integrating SPARK–ICA with multimodal neuroimaging data, such as EEG–fMRI or MEG–fMRI, could help bridge spatial‐frequency findings with electrophysiological signatures, enabling more direct neurobiological interpretation of fine‐scale spatial dynamics.

Sixth, future work could explore subject‐adaptive or hierarchical group models to relax the assumption of shared latent spatial‐frequency structure across individuals, potentially improving sensitivity to personalized network organization and rare pathological patterns.

Finally, the frequency‐resolved dynamic features derived from SPARK–ICA may serve as informative biomarkers for clinical prediction, subtyping, or treatment response, particularly when combined with machine learning approaches. Investigating their utility in classification and prognosis represents a promising translational direction.

In summary, SPARK–ICA advances dynamic fMRI analysis by explicitly incorporating spatial frequency into the study of network dynamics, offering a richer and more nuanced characterization of brain connectivity alterations. The framework may therefore facilitate further investigation of fine‐scale spatiotemporal brain organization and its disruption across neuropsychiatric disorders.

## Funding

The authors have nothing to report.

## Conflicts of Interest

The authors declare no conflicts of interest.

## Supporting information


**Figure S1: Supplementary** PCA scree plot used for model order determination for NeuroMark‐49.

## Data Availability

Data sharing not applicable to this article as no datasets were generated or analysed during the current study.
