# Supplementary material for: SPARK‐ICA: Guided Frequency‐Resolved Dynamic Spatial ICA Reveals Fine‐Scale fMRI Network Dynamics
Source: Hum Brain Mapp. 2026 Nov 2;47(16):e70662. doi: 10.1002/hbm.70662 (PMC13725785; doi:10.1002/hbm.70662)
Supplement: Supplementary file 1 [file HBM-47-e70662-s001.docx]

# Supplementary Figure S1. PCA scree plot used for model order determination for NeuroMark-49.

The plot shows the eigenvalues obtained from PCA of the NeuroMark-49 input data. The red marker indicates the selected model order based on the 95% cumulative explained-variance criterion, corresponding to six components. This model order was subsequently used for the group-level ICA decomposition of the NeuroMark-49 network. NeuroMark-49 is shown as an illustrative example; the same model-order selection procedure was applied independently to the other analyzed NeuroMark networks.
